# The Association Between Plant-Based Diet Indices and Obesity and Metabolic Diseases in Chinese Adults: Longitudinal Analyses From the China Health and Nutrition Survey

**DOI:** 10.3389/fnut.2022.881901

**Published:** 2022-06-20

**Authors:** Bo Chen, Jingjing Zeng, Minghui Qin, Wenlei Xu, Zhaoxia Zhang, Xiaying Li, Shaoyong Xu

**Affiliations:** ^1^Center for Clinical Evidence-Based and Translational Medicine, Xiangyang Central Hospital, Affiliated Hospital of Hubei University of Arts and Science, Xiangyang, China; ^2^Department of Endocrinology, Xiangyang Central Hospital, Affiliated Hospital of Hubei University of Arts and Science, Xiangyang, China; ^3^Department of Traditional Chinese Medicine, Xiangyang Central Hospital, Affiliated Hospital of Hubei University of Art and Science, Xiangyang, China; ^4^Department of Endocrinology, Daxing Hospital, Xi'an, China; ^5^College of Medicine, Wuhan University of Science and Technology, Wuhan, China

**Keywords:** obesity and metabolic diseases, longitudinal analyses, China Health and Nutrition Survey, healthy plant-based diet index, plant-based diet index

## Abstract

**Background:**

A wide range of health benefits are associated with consuming a diet high in plant-based foods. Diet quality can be accurately assessed using plant-based diet indices, however there is inadequate evidence that plant-based diet indices are linked to obesity, hypertension, and type 2 diabetes (T2D), especially in Chinese cultures who have traditionally consumed plant-rich foods.

**Methods:**

The data came from the China Nutrition and Health Survey. Overall, 11,580 adult participants were enrolled between 2004 and 2006 and followed up until 2009 or 2015 (follow-up rate: 73.4%). Dietary intake was assessed across three 24-h recalls, and two plant-based dietary indices [overall plant-based diet indice (PDI) and healthy plant-based diet indice (hPDI)] were calculated using China Food Composition Code and categorized into quintiles. The study's endpoints were overweight/obesity, hypertension, and T2D. The Hazard ratio (HR) and dose-response relationship were assessed using the Cox proportional risk model and restricted cubic splines. The areas under the curve of the receiver operating characteristic curve analyses were used to evaluate the predictive performance of the PDI and hPDI.

**Results:**

During the median follow-up period of more than 10 years, 1,270 (33.4%), 1,509 (31.6%), and 720 (11.5%) participants developed overweight / obesity, hypertension, and T2D, respectively. The higher PDI score was linked with a reduced risk of overweight/obesity [HR: 0.71 (95% CI: 0.55–0.93), *P*-trend <0.001], hypertension [HR: 0.63 (95% CI: 0.51–0.79), *P*-trend <0.001], and T2D [HR: 0.79 (95% CI: 0.72–0.87), *P*-trend <0.001]. The hPDI score was inversely associated with overweight/obesity [HR: 0.79 (95% CI: 0.62–0.98), *P*-trend = 0.02] and T2D [HR: 0.84 (95% CI: 0.75–0.93), *P*-trend = 0.001]. In the aged <55-year-old group, subgroup analysis indicated a significant negative association between PDI/hPDI and overweight/obesity, hypertension, and T2D. There was no significant difference in the areas under the curve of the fully adjusted obesity, hypertension, and diabetes prediction models between PDI and hPDI.

**Conclusion:**

The PDI and hPDI scores were very similar in application in Chinese populations, and our findings highlight that adherence to overall plant-based diet index helps to reduce the risk of T2D, obesity, and hypertension in Chinese adults who habitually consume plant-based foods, especially for those aged <55 year. Further understanding of how plant-based diet quality is associated with chronic disease will be needed in the future, which will help develop dietary strategies to prevent diabetes, hypertension, and related chronic diseases.

## Introduction

The prevalence of obesity, type 2 diabetes (T2D), and hypertension, which are three key risk factors for cardiovascular disease, is increasing globally. It is estimated that T2D affects more than 370 million people and uncontrolled hypertension more than 970 million people worldwide (1), while the global prevalence of overweight and obesity has increased from 20% in 1975 to 40% in 2016 (2). Obesity, T2D, and hypertension constitute the leading causes of death from non-communicable diseases.

The Global Burden of Disease study identified diet as a major modifiable risk factor for non-communicable diseases morbidity and mortality (3). Food group analyses are easier to interpret and translate into recommendations for the primary prevention of obesity, hypertension, and T2D than those of nutrients or complex dietary patterns. Most studies have focused on whether high adherence to plant-based diets and restricted animal diets is associated with poor health outcomes, finding that individuals on vegetarian diets have good metabolic profiles [e.g., lower body mass index (BMI), lower blood pressure, and lower fasting glucose] (4–7). However, extreme dietary changes (e.g., the complete exclusion of animal-based foods) may be difficult to adopt and adhere to over the long term when translating research findings into public health and clinical applications. Moreover, different plant foods are associated with different health outcomes. In particular, less nutrient-dense plant foods (e.g., sugary drinks and salt) are associated with a higher risk of obesity, T2D, and hypertension.

In this context, “plant-based diet indices” (PDIs), which assess the degree of dietary adherence in higher plant-based foods and lower animal-based foods, form a new indicator that has recently been introduced. The “healthy plant-based diet index” (hPDI) measures higher adherence to healthy plant-based foods (e.g., fruits, vegetables, whole grains, nuts, legumes, tea, and coffee) and lower adherence to unhealthy plant-based foods (e.g., refined grains and high-sugar foods) and animal-based foods.

A few studies have used PDIs to assess plant food and animal food intake and considered the health status of plant foods. These studies have focused on hypertension (8), diabetes mellitus (9), weight change (10), chronic kidney disease (11), and metabolic syndrome (12). However, evidence on the association between PDIs and the risk of obesity, hypertension, and T2D is still scant. More importantly, these studies have been performed mainly in Western adult populations living in developed countries (i.e., the United States and Europe). Only two prospective studies on PDIs have been conducted in Asian populations (12, 13). Considering the heterogeneity found in the quantity and quality of diets in different populations and its effects on the health statuses (14, 15), these associations may require further analysis in Asian populations that traditionally consume diets rich in plant foods.

The aim of this study was to assess the association between the PDI and the hPDI and overweight/obesity, hypertension, and T2D using data from the China Health and Nutrition Survey (CHNS).

## Materials and Methods

### Study Design and Participants

The CHNS is an open, prospective, population-based, longitudinal study (16) that aims to explore the impact of the socio-economic transformation of Chinese society on the nutritional and health status of the Chinese population. Launched in 1989 and followed up on in 1991, 1993, 1997, 2000, 2004, 2006, 2009, 2011, and 2015, the CHNS uses a multi-stage random sampling strategy to draw samples from 15 provinces with different demographic, geographic, economic, and public resources (17). The scientific rationale and design of the CHNS have been reported previously (18). Adult participants in the 2004 and 2006 surveys were included in this report because the China Food Composition Code (2002/2004 edition) began to be used in 2004(19, 20), and we used the 2015 survey as the primary study endpoint time, while the diabetes outcome assessment also considered the 2009 glucose/glycated hemoglobin (HbA1c) index. The Institutional Review Board of the University of North Carolina at Chapel Hill and the National Institute of Nutrition and Health of the Chinese Center for Disease Control and Prevention approved the investigation (No. 201524). All participants gave their informed consent before the study. This study is reported according to the Strengthening the Reporting of Observational Studies in Epidemiology (STROBE) guidelines.

**Figure 1:**
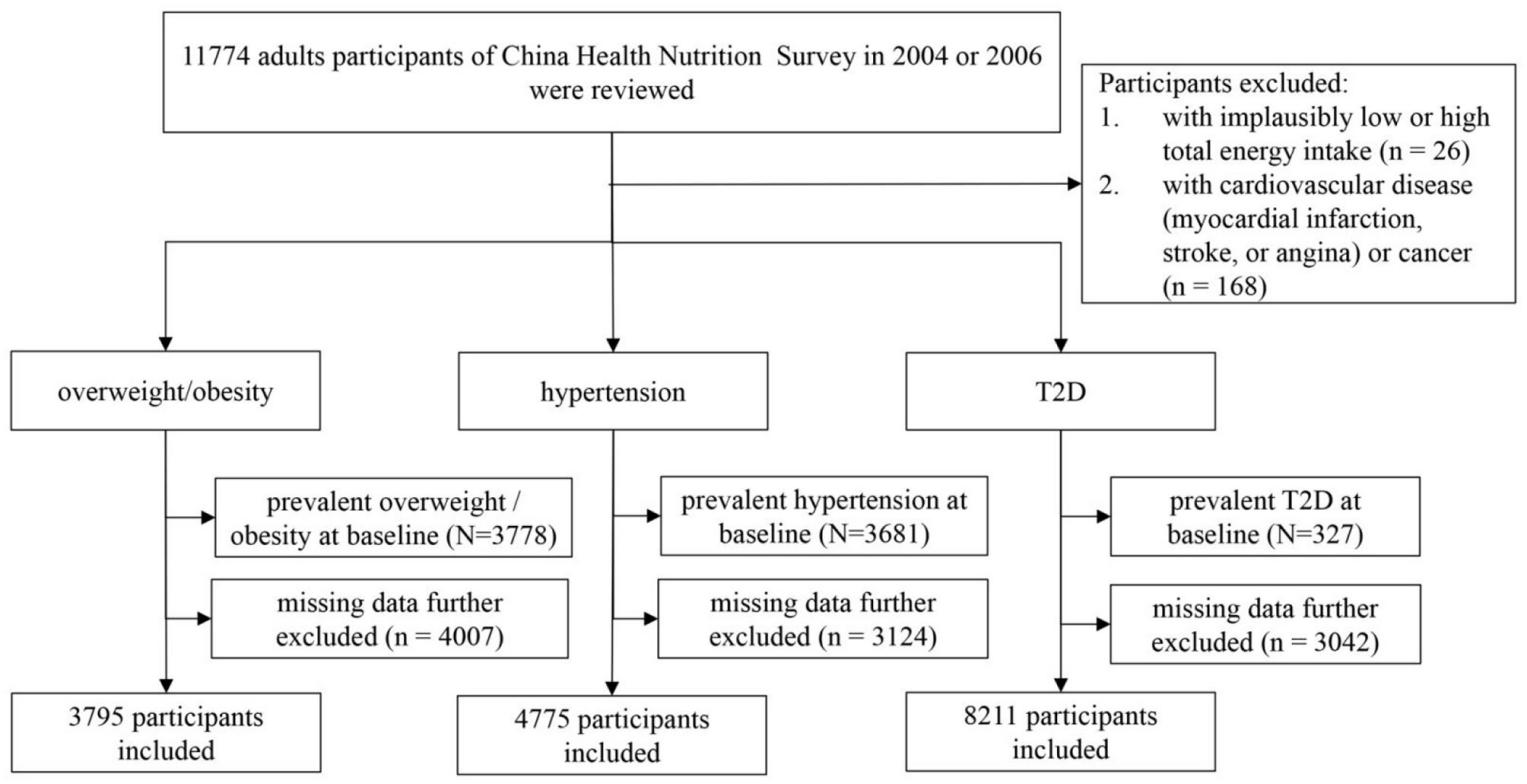
Participant flow diagram.

From the total number of adult participants at baseline (*n* = 11,774), we excluded 26 with extremely low or high total energy intakes (<500 kcal/day or >5,000 kcal/day, respectively) and 168 participants with cardiovascular disease (i.e., myocardial infarction, stroke, or angina) or cancer because a diagnosis of chronic disease may prompt individuals to change their dietary behavior. A total of 8,503 individuals were tracked to 2009 or 2015 (follow-up rate: 73.4%). We excluded 3,778, 3,681, and 327 participants who were overweight or had obesity, had hypertension, and had diabetes at baseline, respectively. Finally, we excluded 4,007 overweight/obese, 3,124 hypertensives, and 3,042 diabetic individuals with missing outcomes, respectively. In the final analysis sample, a total of 3,795 individuals in Sample 1 were used to study the association between PDIs and overweight/obesity, a total of 4,775 individuals in Sample 2 were used to study the association between PDIs and hypertension, and a total of 8,211 individuals in Sample 3 were used to study the association between PDIs and T2D (Figure 1).

### Dietary Assessment

At the time of the baseline (2004 or 2006) survey, dietary assessment in the CHNS was performed by trained researchers during the same period (2 weekdays and 1 weekend day) utilizing three consecutive 24-h individual-level food recalls and household-level food inventory weighing (see Supplementary S1 **Text for the Dietary Questionnaire**). Detailed information on the dietary assessment has been previously published (18). In brief, all available food (purchased, stored, or home produced) was weighed daily on a digital scale (minimum 1 g, maximum 3 kg). Household food consumption is estimated by examining the changes in household food stocks and waste. With the help of the food model, individual dietary intake was estimated by asking each household member to report the type, quantity, time, and place of consumption of all foods consumed at home and outside of the home the previous day. For each dish prepared at home, the amount of food consumed by each individual was estimated based on the ratio of household food consumption to individual consumption. If there were significant differences in food consumption at the household level and individual level, the interviewer returned to the household and the individual in question to record further details regarding the individual's food consumption. Dietary intake of cereals, meat, vegetables, and fruits was estimated for each individual based on the mean of three 24-h dietary recalls.

To ensure the stability of the dietary data, we used average year data for 2004 and 2006 as the baseline dietary assessment. When participants participated in only one of the survey years, the current year's dietary intake could be utilized as the baseline assessment.

We calculated two plant-based diet indices (PDI, hPDI) based on average 24-h dietary assessment data. The calculation of each dietary index and the differences between the indices have been described in detail in previous studies (8, 9, 12). Briefly, referring to the China Food Composition Code (2002/2004 edition) and the classification of food items in the aforementioned studies, all foods were classified into 17 food groups (Supplementary Table S1) in this study. These food groups were classified as healthy plant foods (whole grains, fruits, vegetables, nuts, legumes, tea and coffee, and vegetable oils), less healthy plant foods (refined grains, potatoes, sugary drinks, sweets and desserts, and preserved foods), and animal foods (animal fats, dairy products, eggs, fish or seafood, and meat). Considering the specificity of the Chinese diet, we added the category “fermented foods” and deleted the category “miscellaneous animal foods” (considering that almost none of our subjects reported consuming them).

Considering that residuals were independent of food consumption, this study adjusted for total energy intake by using the residual method and ranked participants by quintile for each food group (21) to give a positive or a negative score. For the PDI, all plant foods were scored positively; for the hPDI, only healthy plant foods were scored positively. For a positive score, individuals in the fifth quartile scored five points and individuals in the first quartile scored one point. For negative scores, individuals in the fifth quartile scored one point and individuals in the first quartile scored five points. The scores of 17 food groups for each participant were then summed to obtain a theoretical range of indices ranging from 17 to 85, and the overall index was divided into quintiles for analysis (22), with higher quartiles representing better adherence.

### Measurement

A structured questionnaire was used to collect participant information including demographic characteristics (age, gender, and education), lifestyle (total energy intake, physical activity, smoking status, and alcohol intake), general health status (diabetes and hypertension), and disease history (myocardial infarction, stroke, angina, and cancer). Participants were asked to wear only light clothing and no shoes when measuring their height and weight and to measure their height to the nearest 0.1 cm using a SECA tape measure and measure their weight to the nearest 0.1 kg using a calibrated balance scale: body mass index (BMI) (kg/m^2^) = weight (kg)/height (m)^2^. Waist circumference was measured using a SECA tape measure at the narrowest point between the lowest rib of the ilium and the uppermost margin to the nearest 0.1 cm. After 5 min of sedation, blood pressure was monitored using a mercury sphygmomanometer on three consecutive occasions, each 3–5 min apart, and the mean of the three systolic and diastolic blood pressure measurements was counted in the analysis.

Blood samples for 2009 were collected according to strict quality control standards specifications, and after a minimum 12-h overnight fast, blood samples (12 mL) were collected by venipuncture in the morning and transferred to a local hospital for processing within 2 h of collection. All blood samples were analyzed at the Beijing National Central Laboratory (Medical Laboratory Accreditation Certificate: ISO 15189:2007) (23). Fasting glucose determination and routine blood tests were performed at the local hospital using the glucose oxidase-phenol and 4-amino benzoquinone method (Randox Laboratories Ltd., Crumlin, Co. Antrim, UK). HbA1c was determined by high-performance liquid chromatography (HLC-723 G7 analyzer; Tosoh Corp., Tokyo, Japan).

### Assessment of Covariates

We classified education levels into four categories: elementary school and below, middle school, high school, and college and above. We calculated the daily metabolic equivalent task for each participant by calculating the type and intensity of physical activity (24). Smokers were defined as subjects who smoked an average of one or more cigarettes per day for at least 6 months before the survey and were still smoking in the 1 month before the survey; other subjects were categorized as non-smokers. Alcohol drinkers were defined as those who consumed beer, liquor, or other alcoholic beverages during the past year; other subjects were classified as non-drinkers. The model was adjusted for age, total energy intake, physical activity, and BMI as continuous variables.

### Ascertainment of Overweight/Obesity, Hypertension, and T2D

Based on the overweight and obesity criteria recommended by the Chinese Working Group on Obesity, this study defined overweight/obesity as BMI ≥24 kg/m^2^ (25, 26).

In the CHNS, questions about hypertension were asked as follows: (1) “Has a doctor told you that you have hypertension?” If so, (2) “How many years have you had hypertension?” and (3) “Do you take antihypertensive medication?” hypertension was defined as meeting at least one of the following criteria: (1) systolic blood pressure ≥140 mmHg, (2) diastolic blood pressure ≥90 mmHg, or (3) self-reported as having been diagnosed with hypertension or being on oral antihypertensive medication during follow-up (27).

In the CHNS, the following questions were asked about diabetes: (1) “Has a doctor told you that you have diabetes?” If so, (2) “How old were you when the diabetic disease was diagnosed?” and (3) “Have you had any of the following treatments, such as special diet, weight control, oral medication, insulin injections, herbal medicine, home remedies, qigong, etc.?” Based on the diagnostic criteria of the American Diabetes Association (28), T2D was defined as meeting at least one of the following criteria in the 2009 survey: (1) fasting blood glucose concentration of ≥7.0 mmol/L (126 mg/dL), (2) HbA1c ≥6.5%, or (3) self-reported diagnosis of T2D or on hypoglycemic medication. In 2015, it was defined based on self-reported diabetes or taking hypoglycemic medication. Previous studies have demonstrated that self-reported diabetes is a relatively valid tool for obtaining the diabetes status of Chinese study participants (29).

### Statistical Analyses

Descriptive analyses were reported as the mean ± standard deviation (SD) or median (interquartile range), estimated as the mean ± SD for continuous variables and frequency (percentage) for categorical variables. Statistical differences in disease occurrence between groups were tested by analysis of variance, the Kruskal–Wallis test, or the chi-square test, respectively.

Following the methodological approach of the database (30), we used Spearman correlation analysis to explore the relationship between baseline PDI and hPDI. We used Cox proportional hazards models to estimate the association between PDIs and overweight/obesity, hypertension, and diabetes mellitus. The time indicator was the duration of follow-up from baseline (2004 or 2006) to disease onset or the cut-off date. Time-dependent covariates analysis validated the assumption of equal proportional risk. Multiple covariates were selected and adjusted in the three models. In model 1, we adjusted for urban–rural, age, sex, and total energy intake. In model 2, we further adjusted for educational level, physical activity, smoking, and drinking. In model 3, we made additional adjustments for BMI. To test for potential non-linear associations, we tested for linear trends using the median score per quantile of index scores. By using index scores as continuous variables, we estimated the risk of disease for each standard deviation (1 SD) increase in each dietary index and also fitted restrictive cubic splines to the fully adjusted model to further observe the association between plant-based dietary indices and disease. The areas under the curve (AUC) of the receiver operating characteristic curve (ROC) analyses were used to evaluate the predictive performance of the PDI and hPDI. Since some studies have shown that age and sex (31, 32) associations between diet and disease may differ, we further stratified the results by sex and age and investigated whether the PDIs were associated with different sex and age groups. All statistical tests were two-sided and performed using SAS 9.4 (SAS Institute, Cary, NC) and R-4.1.2 (http://www.R-project.org/); *p* < 0.05 was considered statistically significant.

## Results

### Characteristics of Participants

During a median follow-up of 10.76 years (total person-years: 40,845), 1,270 (33.4%) participants became overweight/developed obesity with a mean age of 44.01 ± 12.43 years. During a median follow-up of 10.28 years (total person-years: 49,127), 1,509 (31.6%) participants developed hypertension with a mean age of 48.79 ± 11.55 years. During a median follow-up of 10.18 years (total person-years: 83,633), 720 (8.7%) participants developed T2D with a mean age of 53.58 ± 12.14 years. In the disease prevalence overlap, 83 (11.5%) participants with T2D also suffered from being overweight/obesity, and 374 (24.8%) participants with hypertension also suffered from being overweight/obesity. In addition, 149 (1.8%) participants had both T2D and hypertension, and 28 (0.3%) participants had all three diseases simultaneously.

Comparisons of baseline characteristics between those who did and did not develop T2D, hypertension, and overweight/obesity are shown in Table 1. Age, BMI, waist circumference, and blood pressure significantly affect the incidence of all diseases. In addition, education and drinking affect the incidence of diabetes. Total energy intake and animal food intake affect the incidence of obesity. Hypertension has the most influencing factors; in addition to common factors, it also includes urban and rural areas, less healthy plant foods, animal foods, smoking, drinking, and education level.

**Table 1:** Baseline characteristics of cohort by incident overweight/obesity, T2DM, or hypertension.

Characteristics*	**No overweight/obesity**	**New overweight/obesity**	***p***	**No hypertension**	**New hypertension**	***p***	**No T2DM**	**New T2DM**	***p***
Number	2,525	1,270		3,266	1,509		8,211	720	
Age (years)	46.3 ± 13.4	44.0 ± 12.4	<0.001	42.4 ± 12.7	48.7 ± 11.5	<0.001	46.1 ± 14.2	53.5 ± 12.1	<0.001
Male, *n* (%)	1,185 (47.4)	623 (49.5)	0.21	1,495 (46.1)	730 (48.6)	0.09	3,594 (48.3)	366 (50.2)	0.29
Rural, *n* (%)	1,848 (73.2)	945(74.4)	0.42	2,320 (71.0)	1,152 (76.3)	<0.001	5,185 (69.3)	491 (67.1)	0.22
Total energy intake (kcal/day)	2231.6 ± 597.3	2271.1 ± 653.2	0.03	2,244.3 ± 605.1	2259.1 ± 642.2	0.44	2,203.0 ± 571.2	2214.2 ± 640.7	0.56
Educational level, *n* (%)			0.09			<0.001			<0.001
Primary school or below	1,154 (46.2)	541 (43.1)		1,321 (40.7)	732 (48.8)		3,284 (44.1)	384 (52.7)	
Junior high school	851 (34.1)	458 (36.5)		1,196 (36.9)	489 (32.6)		2,468 (33.2)	188 (25.8)	
Senior high school	299 (11.9)	172 (13.7)		435 (13.4)	186 (12.4)		979 (13.1)	87 (11.9)	
College and above	194 (7.7)	85 (6.8)		292 (9.0)	93 (6.2)		700 (9.4)	69 (9.4)	
Physical activity (MET/day)	184.6 ± 124.2	192.3 ± 127.8	0.15	185.7 ± 124.5	181.0 ± 128.7	0.33	180.6 ± 125.1	173.2 ± 126.1	0.28
Smoking, *n* (%)	868 (34.7)	416(33.1)	0.31	1,010 (31.1)	519 (34.6)	0.01	2,437 (32.7)	253 (34.8)	0.24
Alcohol drinking, *n* (%)	840 (33.6)	429 (34.1)	0.71	1,028 (31.6)	552 (36.7)	<0.001	2,462 (33.1)	271 (37.2)	0.02
BMI (kg/m^2^)	20.9 ± 1.9	22.8 ± 2.1	<0.001	22.3 ± 2.9	23.6 ± 3.3	<0.001	22.86 ± 3.2	25.3 ± 3.7	<0.001
Systolic BP (mm Hg)	117.4 ± 15.7	118.9 ± 16.4	0.01	114.0 ± 12.2	121.9 ± 13.6	<0.001	120.5 ± 17.3	130.1 ± 20.3	<0.001
Diastolic BP (mm Hg)	75.9 ± 10.2	77.9 ± 10.8	<0.001	74.6 ± 8.6	79.0 ± 9.1	<0.001	78.1 ± 10.7	82.7 ± 12.1	<0.001
Waist circumference (cm)	75.4 ± 7.6	80.3 ± 7.8	<0.001	78.3 ± 8.9	82.3 ± 9.4	<0.001	80.2 ± 9.5	87.3 ± 10.1	<0.001
**Food groups**
Healthy plant foods (g/day)	540.0 ± 245.7	546.8 ± 274.8	0.41	546.1 ± 265.3	551.9 ± 266.0	0.48	564.8 ± 273.2	547.1 ± 252.8	0.22
Less-healthy plant foods (g/day)	433.2 ± 179.8	435.8 ±200.7	0.64	435.9 ± 191.2	423.2 ± 195.8	0.03	418.7 ± 187.4	426.5 ± 183.8	0.20
Animal foods (g/day)	169.7 ± 131.0	154.3 ± 129.7	<0.001	168.0 ± 131.9	156.2 ± 128.2	<0.001	173.9 ± 126.1	176.2 ± 138.3	0.41
Overall plant-based diet indice	19.3 ± 5.9	18.4 ± 5.7	<0.001	19.4 ± 6.1	18.7 ± 5.8	<0.001	20.2 ± 6.1	19.9 ± 6.1	0.15
Healthy plant-based diet indice	23.4 ± 14.8	23.2 ± 15.7	0.54	19.0 ± 6.9	18.7 ± 6.8	0.17	20.3 ± 7.1	19.7 ± 7.0	0.04

* *Descriptive analyses of continuous variables were analyzed by means ± Standard deviations (SD) or medians (interquartile range) were estimated mean ± SD, and categorical variables were described by number (percentage). Analysis of variance (ANOVA) or Kruskal–Wallis test was used for continuous variables, and chi square test was used for categorical variables. BMI, body mass index; BP, blood pressure*.

In the present study, PDI and hPDI showed the highest Spearman correlation for overweight or obesity (ρ = 0.76), and Spearman correlation coefficients were 0.72 and 0.73 for T2D and hypertension.

### Food Characteristics

In the overweight/obesity, hypertensive, and T2D populations, the highest quintile of PDI and hPDI had higher intakes of fruits, vegetables, nuts, legumes, potatoes, and salty foods and lower total intakes of meat (Supplementary Table S3). In the PDI, the highest quintile consumed on average 118–130 g of fruit and 377–380 g of vegetables per day, while the lowest quintile consumed on average 9.9–10.2 g of fruit and 320–362 g of vegetables per day. The highest quintile in the hPDI consumed on average 111.2–137.0 g of fruit and 348.9–375.1 g of vegetables per day, while in the lowest quintile, 5.2–7.5 g of fruit and 353.4–358.7 g of vegetables per day were consumed.

### Risk of Overweight/Obesity by Baseline PDI and hPDI Quartiles

**Figure 2:**
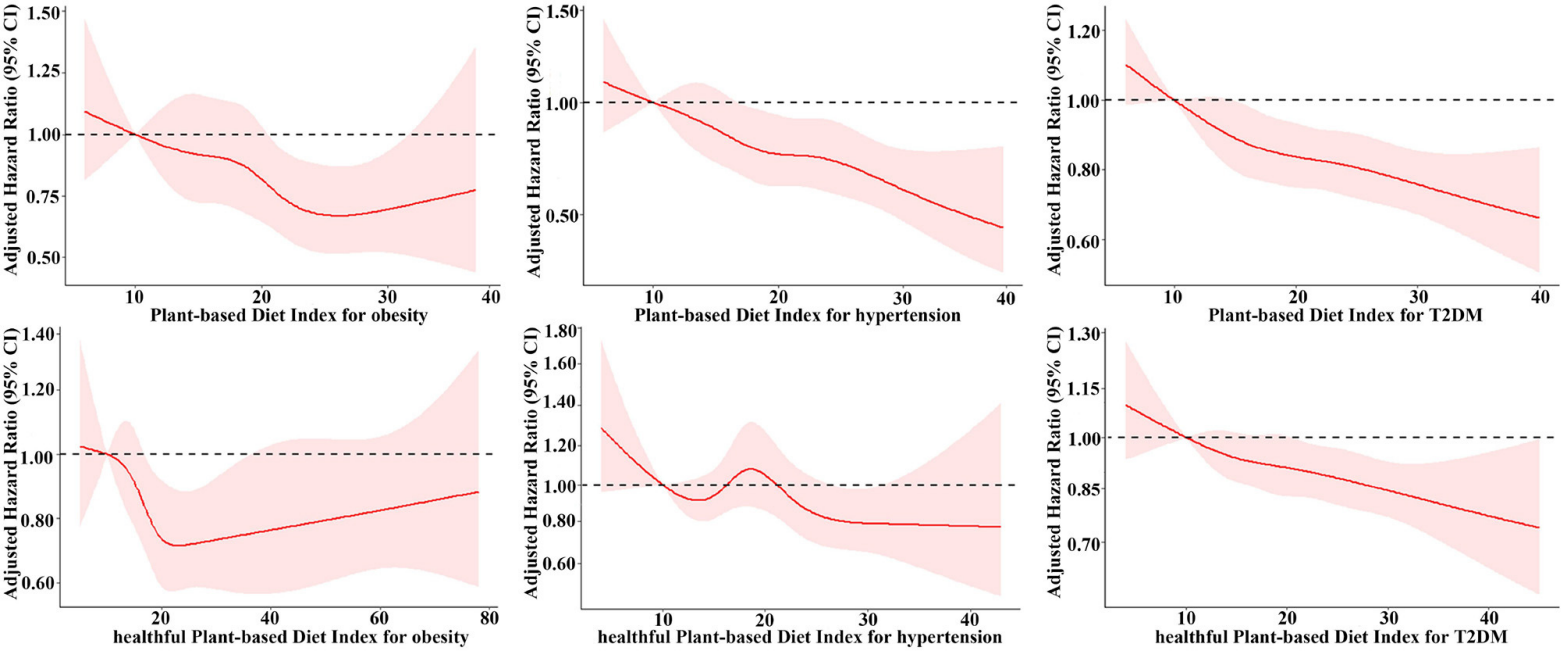
Adjusted hazard ratios and 95% confidence intervals for incident overweight/obesity, hypertension and type 2 diabetes according to the continuous PDI and hPDI. Adjusted for age, urban and rural, sex, total energy intake, education, physical activity, smoking status, alcohol drinking, baseline systolic and diastolic blood pressure, and BMI. BMI, body mass index; T2DM, type 2 diabetes mellitus.

**Figure 3:**
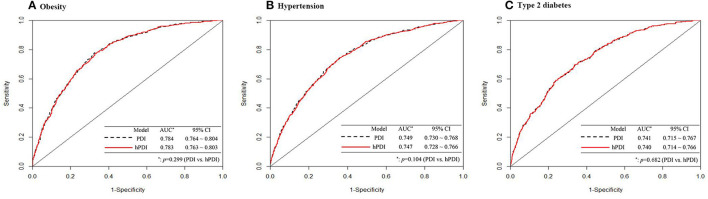
**(A–C)** Receiver-operating characteristic curves for PDI and hPDI predicting incident overweight/obesity, hypertension and type 2 diabetes. Model_PDI_ and Model_hPDI_: Adjusted for age, urban and rural, total energy intake, education, physical activity, smoking status, alcohol drinking, baseline systolic and diastolic blood pressure, and BMI. AUC: area under curve.

Table 2 indicates the incidence of overweight/obesity for individuals stratified by baseline PDI and hPDI quintiles. The trend in the incidence of overweight/obesity across increasing PDI and hPDI quintiles was highly statistically significant for all models (*p* < 0.05). In the multivariate models, the adjusted Hazard ratios (HR) was attenuated but remained significant in the comparison of the highest (Q5) and lowest (Q1) quartiles. When the PDI was modeled consecutively, the risk of overweight/obesity was reduced by 11% for every 1 SD increase in the index, which was statistically significant (95% CI: 0.82–0.97; *p*-trend < 0.001). When hPDI was assessed consecutively, there was a statistically significant difference of a 4% reduction in the risk of overweight/obesity occurrence for every 1 SD increase in the index (95% CI: 0.89–1.00; *p*-trend = 0.02). However, the restricted cubic spline of the association between PDI/hPDI and the risk of occurrence of overweight/obesity showed a U-shaped trend of decreasing and then gradually increasing the risk of overweight/obesity with increasing PDI/hPDI (Figure 2). The full adjustment overweight/obesity prediction model yielded an AUC of 0.784 (95% CI: 0.764–0.804) for PDI and 0.783 (95% CI: 0.763–0.803) for hPDI, with no significant difference (*p* = 0.299) (Figure 3A).

**Table 2:** Prospective associations between plant-based diet indices and incident overweight/obesity, hypertension and type 2 diabetes mellitus^a^.

**Incident overweight/obesity (*****N*** **= 3,795)**							
	**Quintile 1**	**Quintile 2**	**Quintile 3**	**Quintile 4**	**Quintile 5**	**P-trend**	**HR (95%) per 1 SD increase**
**PDI**							
Number of cases	253	342	250	233	192		
Person-years	7,024	10,183	8,027	8,048	7,563		
Incidence density (1,000 person-year)	36.0	33.6	31.1	29.0	25.4		
Model 1	Ref	0.87 (0.74, 1.03)	0.81 (0.68, 0.95)	0.72 (0.61, 0.87)	0.62 (0.61, 0.76)	<0.001	0.85 (0.81, 0.90)
Model 2	Ref	0.83 (0.68, 1.01)	0.77 (0.62, 0.96)	0.65 (0.51, 0.81)	0.59 (0.46, 0.74)	<0.001	0.84 (0.78, 0.90)
Model 3	Ref	0.94 (0.76, 1.18)	0.89 (0.70, 1.11)	0.78 (0.61, 1.01)	0.71 (0.55, 0.93)	<0.001	0.89 (0.82, 0.97)
**hPDI**							
Number of cases	290	272	227	234	247		
Person-years	8,049	8,434	7,914	8,174	82,74		
Incidence density (1,000 person-year)	36.0	32.3	28.7	28.6	29.9		
Model 1	Ref	0.98 (0.75, 1.07)	0.77 (0.65, 0.92)	0.77 (0.65, 0.92)	0.75 (0.63, 0.89)	<0.001	0.94 (0.89, 1.00)
Model 2	Ref	0.89 (0.72, 1.09)	0.66 (0.53, 0.84)	0.72 (0.58, 0.89)	0.71 (0.57, 0.88)	<0.001	0.94 (0.87, 1.01)
Model 3	Ref	0.94 (0.75, 1.18)	0.76 (0.58, 0.98)	0.79 (0.63, 1.00)	0.79 (0.62, 0.98)	0.02	0.96 (0.89, 1.00)
**Incident hypertension (*****N*** **= 47,75)**							
	**Quintile 1**	**Quintile 2**	**Quintile 3**	**Quintile 4**	**Quintile 5**	**P-trend**	**HR (95%) per 1 SD increase**
**PDI**							
Number of events	382	297	268	322	240		
Person-years	106,88	9,447	9,371	10,036	9,585		
Incidence density (1,000 person, year)	35.7	31.4	28.6	32.1	25.0		
Model 1	Ref	0.84 (0.72, 0.97)	0.76 (0.65, 0.89)	0.87 (0.75, 1.01)	0.68 (0.58, 1.81)	<0.001	0.89 (0.84, 0.94)
Model 2	Ref	0.81 (0.66, 0.98)	0.70 (0.57, 0.86)	0.85 (0.70, 1.02)	0.61 (0.49, 0.75)	<0.001	0.86 (0.81, 0.93)
Model 3	Ref	0.83 (0.67, 1.00)	0.73 (0.59, 0.90)	0.91 (0.74, 1.10)	0.63 (0.51, 0.79)	<0.001	0.88 (0.82, 0.94)
**hPDI**							
Number of cases	349	340	255	320	245		
Person-years	10,945	11,101	8,352	10,190	8,539		
Incidence density (1,000 person-year)	31.9	30.6	30.5	31.4	28.7		
Model 1	Ref	0.95 (0.82, 1.11)	0.94 (0.80, 1.06)	0.96 (0.83, 1.12)	0.89 (0.76, 1.05)	0.26	0.95 (0.91, 1.01)
Model 2	Ref	1.04 (0.87, 1.26)	0.99 (0.81, 1.22)	0.93 (0.76, 1.14)	0.84 (0.67, 1.04)	0.07	0.93 (0.86, 0.99)
Model 3	Ref	1.04 (0.86, 1.27)	1.08 (0.87, 1.33)	0.95 (0.78, 1.17)	0.83 (0.66, 1.04)	0.09	0.93 (0.87, 1.00)
**Incident Type 2 diabetes (*****N*** **= 8211)**							
	**Quintile 1**	**Quintile 2**	**Quintile 3**	**Quintile 4**	**Quintile 5**	**P-trend**	**HR (95%) per 1 SD increase**
**PDI**							
Number of cases	149	170	143	137	121		
Person, years	16,140	19,501	16,351	16,013	15,628		
Incidence density (1,000 person-year)	9.2	8.7	8.7	8.6	7.7		
Model 1	Ref	0.81 (0.66, 0.98)	0.70 (0.57, 0.86)	0.85 (0.70, 1.02)	0.61 (0.49, 0.75)	<0.001	0.93 (0.91, 0.95)
Model 2	Ref	0.89 (0.81, 0.98)	0.86 (0.78, 0.95)	0.85 (0.77, 0.94)	0.80 (0.72, 0.88)	<0.001	0.93 (0.90, 0.96)
Model 3	Ref	0.89 (0.81, 0.98)	0.87 (0.78, 1.45)	0.85 (0.76, 0.93)	0.79 (0.72, 0.87)	<0.001	0.93 (0.90, 0.96)
**hPDI**							
Number of cases	156	133	160	165	106		
Person-years	15,803	15,431	18,367	17,164	16,868		
Incidence density (1,000 person-year)	9.9	8.6	8.7	9.6	6.3		
Model 1	Ref	0.96 (0.89–1.03)	0.93 (0.87–1.00)	0.91 (0.84–1.00)	0.91 (0.84–0.98)	<0.001	0.95 (0.93, 0.97)
Model 2	Ref	0.94 (0.86–1.04)	0.91 (0.82–0.99)	0.90 (0.82–0.99)	0.84 (0.76–0.93)	<0.001	0.94 (0.91, 0.97)
Model 3	Ref	0.94 (0.85–1.03)	0.90 (0.81–0.99)	0.90 (0.81–0.99)	0.84 (0.75–0.93)	0.001	0.94 (0.92, 0.98)

*Model 1 was adjusted for age, urban and rural, sex, and total energy intake. Model 2 was adjusted for age, urban and rural, sex, total energy intake, education, physical activity, smoking status, and alcohol drinking. Model 3 was adjusted for age, sex, total energy intake, education, physical activity, smoking status, alcohol drinking, baseline systolic and diastolic blood pressure, and BMI*.

*^a^The quintiles do not have equal sample size because many participants received the same scores*.

*BMI, body mass index; PDI, overall plant-based diet index; hPDI, healthful plant-based diet index; SD, standard deviation*.

### Risk of Hypertension by Baseline PDI and hPDI Quartiles

A higher PDI score was also associated with a reduced risk of developing future hypertension (Table 2). After adjusting for multiple potential covariates, participants in the highest PDI quartile had a 37% lower risk of developing hypertension (HR: 0.63, 95% CI: 0.51–0.79). The linear trend for increasing PDI quartiles remained highly significant in the multivariate model (*p*-trend < 0.001). When the PDI was assessed consecutively, the risk of hypertension was reduced by 12% (95% CI: 0.82–0.94; *p*-trend < 0.001) for every 1 SD increase in the index. There was a strong linear association between a higher PDI score and the incidence of hypertension (Figure 2).

In each hPDI model, no linear trend (*p* > 0.05) was detected in the risk of hypertension development in each quartile of hPDI. In multivariate models, adjusted HRs were all <1, but there was no statistical difference in the highest quartile (Q5) compared with the lowest quartile (Q1). However, when modeling the hPDI consecutively, there was a statistically significant 7% reduction in the risk of hypertension for every 1 SD increase in the index (HR: 0.93, 95% CI: 0.87–1.00). This association was reflected when we visually described the association between hPDI and the incidence of hypertension (Figure 2). The full adjustment hypertension prediction model yielded an AUC of 0.749 (95% CI: 0.730–0.768) for PDI and 0.747 (95% CI: 0.728–0.766) for hPDI, with no significant difference (*p* = 0.104) (Figure 3B).

### Risk of T2D by Baseline PDI and hPDI Quartiles

During 83,633 person-years of follow-up, we recorded 720 cases of diabetes. In the fully adjusted model, a higher PDI/hPDI was significantly associated with a reduced risk of developing T2D (Table 2). A 21% lower risk of developing diabetes was observed in the highest PDI quartile (HR: 0.79, 95% CI: 0.72–0.87, *p*-trend < 0.001) and a 16% lower risk of developing diabetes was observed in the highest hPDI quartile (HR: 0.84, 95% CI: 0.75–0.93, *p*-trend = 0.001). When PDI/hPDI was assessed consecutively, the risk of developing diabetes decreased by 7% (95% CI: 0.90–0.96) and 6% (95% CI: 0.92–0.98) for each 1 SD increase in the index. The association between PDI/hPDI and the risk of developing diabetes was also shown in the restricted cubic spline, indicating that with increasing PDI/hPDI the risk of developing diabetes showed a significant downward trend (Figure 2). The AUC for the full adjustment T2D prediction model was 0.741 (95% CI: 0.715–0.767) for PDI and 0.740 (95% CI: 0.714–0.766) for hPDI, with no significant difference (*p* = 0.682) (Figure 3C).

### Stratified Analyses

We performed a stratified analysis. In the present study, after fully adjusting for relevant confounding, we found that higher PDI and hPDI scores were significantly and negatively associated with the risk of being overweight/having obesity, hypertension, and T2D in the younger population (age <55 years) (*p* < 0.05), while the linear trend of increasing PDI/hPDI quartiles with associated diseases in the younger age group remained highly significant (*p*-trend < 0.02). However, in older people (age ≥ 55 years), there were no statistically significant differences (*p* > 0.05), except for a significant negative association between PDI score and risk of hypertension (*p* < 0.05) (Table 3).

**Table 3:** Hazard ratios (95% CI) for overweight/obesity, hypertension and type 2 diabetes according to quintile of the overall & healthful plant-based diet indices, stratified by age.

	**Quintile 1**	**Quintile 2**	**Quintile 3**	**Quintile 4**	**Quintile 5**	***P*** **-trend^a^**
**Overall plant-based diet index**
**Overweight/obesity**
<55 years	1.00	0.97 (0.76, 1.24)	0.90 (0.71, 1.16)	0.80 (0.62, 1.04)	0.68 (0.51, 0.90)	<0.001
≥55 years	1.00	0.56 (0.31, 0.99)	0.62 (0.34, 1.10)	0.55 (0.28, 1.08)	0.79 (0.44, 1.41)	0.38
**Hypertension**
<55 years	1.00	0.80 (0.63, 1.01)	0.78 (0.63, 0.98)	0.80 (0.62, 1.03)	0.62 (0.49, 0.80)	0.02
≥55 years	1.00	0.87 (0.59, 1.30)	0.82 (0.52, 1.28)	1.10 (0.70, 1.74)	0.58 (0.35, 0.95)	0.08
**Type 2 diabetes mellitus**
<55 years	1.00	0.89 (0.80, 1.01)	0.88 (0.80, 0.98)	0.86 (0.77, 0.95)	0.80 (0.72, 0.89)	<0.001
≥55 years	1.00	0.94 (0.75, 1.17)	0.87 (0.68, 1.11)	0.92 (0.72, 1.17)	0.87 (0.67, 1.12)	0.30
**Healthful plant-based diet index**
Overweight/obesity
<55 years	1.00	0.84 (0.65, 1.09)	0.66 (0.51, 0.86)	0.79 (0.62, 1.01)	0.74 (0.58, 0.95)	0.01
≥55 years	1.00	1.31 (0.73, 2.34)	1.23 (0.65, 2.32)	1.00 (0.51, 1.97)	0.97 (0.54, 1.80)	0.78
**Hypertension**
<55 years	1.00	0.96 (0.77, 1.20)	0.99 (0.77, 1.27)	0.92 (0.73, 1.16)	0.77 (0.60, 0.98)	0.04
≥55 years	1.00	1.20 (0.80, 1.80)	1.34 (0.86, 2.11)	1.08 (0.64, 1.82)	0.87 (0.59, 1.40)	0.61
**Type 2 diabetes mellitus**
<55 years	1.00	0.95 (0.86, 1.05)	0.89 (0.76, 0.99)	0.91 (0.82, 1.01)	0.84 (0.76, 0.94)	0.002
≥55 years	1.00	0.99 (0.81, 1.22)	0.98 (0.77, 1.26)	0.95 (0.74, 1.21)	0.92 (0.72, 1.16)	0.44

*Adjusted for age, urban and rural, sex, total energy intake, education, physical activity, smoking status, alcohol drinking, baseline systolic and diastolic blood pressure, and BMI*.

*^a^p-Value when we assigned the median value to each quintile and entered this as a continuous variable in the model*.

In a gender-stratified analysis, after fully adjusting for relevant confounding, we found that higher PDI and hPDI scores showed significant negative correlations with the risk of being overweight/having obesity in women (*p* < 0.05) and with risk of T2D in both men and women (*p* < 0.05), while we found that only the PDI score showed a significant negative association with the risk of hypertension in both sexes (*p* < 0.05) (Supplementary Table S2).

## Discussion

In this relatively large nationwide cohort study of adults, we found that higher scores on PDIs were associated with a reduced risk of being overweight and having obesity, hypertension, and T2D. Specifically, PDI and hPDI were negatively associated with the risk of having obesity and T2D, and PDI was also negatively associated with the risk of hypertension, and these associations were independent of BMI and other risk factors. Previous prospective studies and meta-analyses have concluded that higher PDI and hPDI scores are negatively associated with lower reductions in weight gain over 4-year intervals (10). Plant-based diets, especially those rich in high-quality plant foods, significantly reduce the risk of T2D (9, 13, 33–35). PDI or hPDI may moderately improve the risk of hypertension (8, 36). Our findings are generally consistent with the current knowledge that adherence to a higher PDI and hPDI score is associated with a reduced risk of becoming overweight/developing obesity, hypertension, and T2D, regardless of the health effects of specific food types.

However, our results are partially inconsistent with the findings of previous studies. First, although we found that higher PDI and hPDI scores were associated with a lower risk of T2D (HR_PDI_: 0.79, 95% CI: 0.72–0.87; HR_hPDI_: 0.84, 95% CI: 0.75–0.93), the association between the hPDI score and the risk of T2D was weaker than that of the PDI. This may be due to different food type compositions. Some studies found no clear association between vegetable and nut intake and diabetes incidence (37), and a much higher intake of vegetables than other healthy plant-based foods were found in our population, which may have somewhat weakened this association. Second, current evidence suggests that high hPDI score significantly reduces the risk of developing hypertension, but the relationship between PDI and the incidence of hypertension has not been established. The US population study concluded that high adherence to both PDI and hPDI significantly reduced the risk of hypertension by 12 to 16% (8). A recent study in a Korean population found that high hPDI score significantly reduced the risk of hypertension by 35% (HR: 0.65, 95% CI: 0.57, 0.75), but PDI was not associated with hypertension (36). Our study contradicted the findings of the Korean population study; it found a significant negative association between high adherence to PDI and hypertension incidence, but no significant association was observed between high adherence to hPDI and hypertension incidence. However, receiver operating characteristic curves show that PDI and hPDI were found to be essentially the same for the AUC of risk of hypertension. Thus, there could be several reasons for this. Firstly, the difference may be influenced by demographic characteristics (socio-demographic characteristics, country, age structure, etc.). The analysis of Korea, which included people aged 40–69 years, and our study, which included people aged ≥18 years, found a significant negative association between high adherence to hPDI and the incidence of hypertension in people aged <55 years (HR_PDI_: 0.62, 95% CI: 0.49–0.80; HR_hPDI_: 0.77, 95% CI: 0.60–0.98). Secondly, the inverse U-shaped distribution of whole grain intake and the W-shaped distribution of meat intake observed in the hPDI quintiles may explain the contradictory findings and non-linear associations for hypertension. In addition, differences in food consumption patterns and methods of obtaining dietary data were associated with the contribution of the PDI and hPDI scores. Of course, the number of available studies on PDI and hPDI is limited, and more studies are needed to analyze these associations.

Notably, although PDI and hPDI were negatively associated with the risk of having obesity, the non-linear association showed a U-shaped trend of decreasing and then gradually increasing the risk of overweight/obesity with increasing PDI/hPDI. Marleen A's study showed that refined grain/high glucose index intake are likely to facilitate weight increasing (38). The U-shaped distribution of refined grain intake observed in the hPDI quintiles and the liner distribution of refined grain intake observed in the PDI quintiles may explain the non-linear association for overweight/obesity in Chinese population.

In the age group analysis, adherence to high PDI and hPDI in people ≥55 years were not found to significantly reduce the risk of developing overweight/obesity, T2D, and hypertension, which may be a result of the progressive decrease in caloric needs of older adults and the progressive increase in the need for nutrients not available in plant foods (e.g., unsaturated fatty acids), with some studies showing that a dietary intake of unsaturated long-chain fatty acids has a protective effect (39). In addition, the risk of diabetes and hypertension is higher in the elderly population than in the younger population because of their physical function. Also, this result is consistent with the results of the Nurses' Health Study 2 data from Ambika Satija et al.'s study, but not with the pooled results from that study (9). Some factors, including differences in dietary patterns across populations, the methods used to obtain dietary data, and the calculation methods used to develop these indices, may have determined these differences. In the gender analysis, women adhered to both the high PDI and hPDI in favor of a lower risk of obesity. This may be because female vegetarians have significantly higher adiponectin levels than non-vegetarians, and studies have shown that lipocalin has protective metabolic effects that reduce inflammation and endothelial dysfunction, which in turn reduces the risk of being overweight/having obesity (40, 41).

Overall, higher PDI and hPDI scores are associated with a decreased risk of overweight/obesity and the development of their metabolic diseases. Our analysis of the correlation and concordance between PDI and hPDI showed several common components between these indices, with higher scores on these indices implying higher intakes of fruits, vegetables, whole grains, nuts, and legumes and lower intakes of red and processed meats. PDI and hPDI were associated with a similar degree of reduced risk of developing chronic diseases. In addition, some studies have shown no significant association between the consumption of vegetables, fruits, and nuts in a healthy plant-based diet and the risk of developing T2D (37), which may have somewhat weakened the protective effect of hPDI on chronic disease. Taken together, these findings support the usual dietary recommendations that recommend a higher intake of plant foods (e.g., whole grains, fruits, vegetables, nuts, and legumes) and a lower intake of animal foods (especially red meat and processed meat), which are captured by the PDI and hPDI.

Our results regarding the association between plant-based diets and overweight/obesity, T2D, and hypertension are biologically plausible when considering the macronutrient and micronutrient composition of plant-based dietary patterns. Those adhering to a higher hPDI consumed higher amounts of protein (12.2–14.5% of total energy), fiber, and potassium compared to those in the lowest quartile. Randomized controlled trials have reported that higher dietary protein and fiber intake are associated with lower systolic and diastolic blood pressure (42). Similarly, a higher potassium intake is believed to lower blood pressure through vasodilation and vascular homeostasis (43). In addition, plant-based diets may also reduce obesity and T2D through several mechanisms (44, 45). Such diets are rich in micronutrients, such as dietary fiber, antioxidants, unsaturated fatty acids, and magnesium, and low in saturated fat. Epidemiological and clinical studies have shown that highly viscous and soluble fiber, lower levels of saturated fat, and lower caloric content have beneficial effects on improving long-term glucose metabolism and reducing energy intake (46).

The strength of our study is that the PDIs we used accounted for dietary changes in the consumption of plant-based and animal-based foods in an integrated manner as compared to previous studies that used single nutrients or single food groups alone. We found that high adherence to plant-based diet scores was effective in reducing the risk of common non-communicable diseases (overweight/obesity, diabetes, and hypertension). This is consistent with previous analyses of individual foods and the risk of weight change, diabetes, and hypertension development in these cohorts (4–7). Another major strength of our study is the detailed collection of dietary intake data, which were collected through repeatedly validated 24-h dietary records based on an extensive database containing 6,900 food items. This allowed us to examine in detail the association between different types of food groups and various chronic diseases (8).

Nonetheless, certain limitations should be recognized. First, although our model adjusts for a variety of potential confounders, residual confounding by unavailable diets, certain medical condition variables, or metabolic factors may persist. Second, participants were categorized into quintiles of the plant-based diet score based on the intake distribution. All of these metrics limit inferences about the absolute intake of animal or plant foods associated with a lower risk of obesity, T2D, and hypertension. Finally, due to database limitations, the 9–11-year time frame is relatively short when looking at total life expectancy, and the young adults in this cohort are unlikely to develop diabetes and hypertension in the next 9–11 years but may still have a higher risk of future diabetes and hypertension, so a longer time frame may be needed for future confirmation.

## Conclusion

The PDI and hPDI scores were very similar in the Chinese population in that they scored positively for fruits, vegetables, whole grains, nuts, and legumes but had the opposite scores for all animal products. Our findings emphasize that adherence to a whole plant-based diet helps reduce the risk of T2D, obesity, and hypertension in Chinese adults, particularly for those <55 years of age. Our findings support a shift toward emphasizing plant-based diets to improve health outcomes. Further understanding of how the quality of plant-based diets is associated with chronic disease will be needed in the future, which will help develop dietary strategies to prevent diabetes, hypertension, and related chronic diseases.

## Data Availability Statement

The datasets presented in this study can be found in online repositories. The names of the repository/repositories and accession number(s) can be found below: Our study relied on data from CHNS. The datasets using during the current study are available at https://www.cpc.unc.edu/projects/china.

## Ethics Statement

The studies involving human participants were reviewed and approved by the Institutional Review Board of the University of North Carolina at Chapel Hill and the National Institute of Nutrition and Health of the Chinese Center for Disease Control and Prevention approved the investigation (No. 201524). The patients/participants provided their written informed consent to participate in this study.

## Author Contributions

BC, JZ, MQ, and SX conceived and designed the study. BC and JZ analyzed the data. BC and MQ wrote the first draft of the manuscript. BC, JZ, MQ, WX, ZZ, XL, and SX contributed to the writing of the manuscript and agreed with the manuscript's results and conclusions. SX helps in funding acquisition. All authors have read and confirm that they meet, ICMJE criteria for authorship.

## Funding

This study was partly supported by the Young Talents Project of Hubei Provincial Health Commission, China (Grand number: WJ2021Q012), Science and Technology Research Key Project of Education Department of Hubei Province, China (Grand number: D20212602), Sanuo Diabetes Charity Foundation, China, and Xiangyang Science and Technology Plan Project, China (Grand number: 2019ZD12).

## Conflict of Interest

The authors declare that the research was conducted in the absence of any commercial or financial relationships that could be construed as a potential conflict of interest.

## Publisher's Note

All claims expressed in this article are solely those of the authors and do not necessarily represent those of their affiliated organizations, or those of the publisher, the editors and the reviewers. Any product that may be evaluated in this article, or claim that may be made by its manufacturer, is not guaranteed or endorsed by the publisher.

## References

[1] Danaei G Finucane MM Lin JK Singh GM Paciorek CJ Cowan MJ. National, regional, and global trends in systolic blood pressure since 1980: systematic analysis of health examination surveys and epidemiological studies with 786 country-years and 5.4 million participants. Lancet. (2011) 377:568–77. 10.1016/S0140-6736(10)62036-3 21295844

[2] Sung H Siegel RL Torre LA Pearson-Stuttard J Islami F Fedewa SA. Global patterns in excess body weight and the associated cancer burden. CA Cancer J Clin. (2019) 69:88–112. 10.3322/caac.21499 30548482

[3] GBD 2017 Diet Collaborators. Health effects of dietary risks in 195 countries, 1990–2017: a systematic analysis for the Global Burden of Disease Study 2017. Lancet. (2019) 393:1958–72. 10.1016/S0140-6736(19)30041-8 30954305 PMC6899507

[4] Haider LM Schwingshackl L Hoffmann G Ekmekcioglu C. The effect of vegetarian diets on iron status in adults: a systematic review and meta-analysis. Crit Rev Food Sci Nutr. (2018) 58:1359–74. 10.1080/10408398.2016.1259210 27880062

[5] Oussalah A Levy J Berthezène C Alpers DH Guéant JL. Health outcomes associated with vegetarian diets: an umbrella review of systematic reviews and meta-analyses. Clin Nutr. (2020) 39:3283–307. 10.1016/j.clnu.2020.02.037 32204974

[6] Lee KW Loh HC Ching SM Devaraj NK Hoo FK. Effects of vegetarian diets on blood pressure lowering: a systematic review with meta-analysis and trial sequential analysis. Nutrients. (2020) 12:1604. 10.3390/nu12061604 32486102 PMC7352826

[7] Huang RY Huang CC Hu FB Chavarro JE. Vegetarian diets and weight reduction: a meta-analysis of randomized controlled trials. J Gen Intern Med. (2016) 31:109–16. 10.1007/s11606-015-3390-7 26138004 PMC4699995

[8] Kim H Rebholz CM Garcia-Larsen V Steffen LM Coresh J Caulfield LE. Operational differences in plant-based diet indices affect the ability to detect associations with incident hypertension in middle-aged US adults. J Nutr. (2020) 150:842–50. 10.1093/jn/nxz275 31722418 PMC7138677

[9] Satija A Bhupathiraju SN Rimm EB Spiegelman D Chiuve SE Borgi L. Plant-based dietary patterns and incidence of type 2 diabetes in US men and women: results from three prospective cohort studies. PLoS Med. (2016) 13:e1002039. 10.1371/journal.pmed.1002039 27299701 PMC4907448

[10] Satija A Malik V Rimm EB Sacks F Willett W Hu FB. Changes in intake of plant-based diets and weight change: results from 3 prospective cohort studies. Am J Clin Nutr. (2019) 110:574–82. 10.1093/ajcn/nqz049 31127828 PMC6735841

[11] Kim H Caulfield LE Garcia-Larsen V Steffen LM Grams ME Coresh J. Plant-based diets and incident CKD and kidney function. Clin J Am Soc Nephrol. (2019) 14:682–91. 10.2215/CJN.12391018 31023928 PMC6500948

[12] Kim H Lee K Rebholz CM Kim J. Plant-based diets and incident metabolic syndrome: results from a South Korean prospective cohort study. PLoS Med. (2020) 17:e1003371. 10.1371/journal.pmed.1003371 33206633 PMC7673569

[13] Chen GC Koh WP Neelakantan N Yuan JM Qin LQ van Dam RM. Diet quality indices and risk of type 2 diabetes mellitus: the Singapore Chinese health study. Am J Epidemiol. (2018) 187:2651–61. 10.1093/aje/kwy183 30165478 PMC6887680

[14] Bhavadharini B Mohan V Dehghan M Rangarajan S Swaminathan S Rosengren A. White rice intake and incident diabetes: a study of 132,373 participants in 21 countries. Diabetes Care. (2020) 43:2643–50. 10.2337/dc19-2335 32873587 PMC7576435

[15] Jacobs S Harmon BE Boushey CJ Morimoto Y Wilkens LR Le Marchand L. A priori-defined diet quality indexes and risk of type 2 diabetes: the Multiethnic Cohort. Diabetologia. (2015) 58:98–112. 10.1007/s00125-014-3404-8 25319012 PMC4258157

[16] Licher S Heshmatollah A van der Willik KD Stricker BHC Ruiter R de Roos EW. Lifetime risk and multimorbidity of non-communicable diseases and disease-free life expectancy in the general population: a population-based cohort study. PLoS Med. (2019) 16:e1002741. 10.1371/journal.pmed.1002741 30716101 PMC6361416

[17] Zhang B Zhai FY Du SF Popkin BM. The China health and nutrition survey, 1989–2011. Obes Rev. (2014) 15(Suppl. 1):2–7. 10.1111/obr.12119 24341753 PMC3869031

[18] He T Wang M Tian Z Zhang J Liu Y Zhang Y. Sex-dependent difference in the association between frequency of spicy food consumption and risk of hypertension in Chinese adults. Eur J Nutr. (2019) 58:2449–61. 10.1007/s00394-018-1797-8 30078091

[19] Yang Y WG Pan X. China Food Composition Code. Beijing: University Medical Press (2002).

[20] Yang Y WG Pan X. China Food Composition Code. Beijing: Peking University Medical Press (2004).

[21] Hu FB Stampfer MJ Rimm E Ascherio A Rosner BA Spiegelman D. Dietary fat and coronary heart disease: a comparison of approaches for adjusting for total energy intake and modeling repeated dietary measurements. Am J Epidemiol. (1999) 149:531–40. 10.1093/oxfordjournals.aje.a009849 10084242

[22] Gómez-Donoso C Martínez-González MÁ Martínez JA Gea A Sanz-Serrano J Perez-Cueto FJA. A provegetarian food pattern emphasizing preference for healthy plant-derived foods reduces the risk of overweight/obesity in the SUN cohort. Nutrients. (2019) 11:1553. 10.3390/nu11071553 31324022 PMC6683267

[23] Li X He T Yu K Lu Q Alkasir R Guo G. Markers of iron status are associated with risk of hyperuricemia among chinese adults: nationwide population-based study. Nutrients. (2018) 10:191. 10.3390/nu10020191 29425155 PMC5852767

[24] Ainsworth BE Haskell WL Whitt MC Irwin ML Swartz AM Strath SJ. Compendium of physical activities: an update of activity codes and MET intensities. Med Sci Sports Exerc. (2000) 32(9 Suppl.):S498–504. 10.1097/00005768-200009001-00009 10993420

[25] Bei-Fan Z and the Cooperative Meta-analysis Group of Working Group on Obesity in China. Predictive values of body mass index and waist circumference for risk factors of certain related diseases in Chinese adults: study on optimal cut-off points of body mass index and waist circumference in Chinese adults. Asia Pacific J Clin Nutr. (2002) 11:S685–93. 10.1046/j.1440-6047.11.s8.9.x 12046553

[26] Gao M Wei YX Lyu J Yu CQ Guo Y Bian Z. The cut-off points of body mass index and waist circumference for predicting metabolic risk factors in Chinese adults. Zhonghua Liu Xing Bing Xue Za Zhi. (2019) 40:1533–40. 10.3760/cma.j.issn.0254-6450.2019.12.006 32062911

[27] Xue Y Shen Q Li C Dai Z He T. The visceral adipose index in relation to incidence of hypertension in Chinese adults: China Health and Nutrition Survey (CHNS). Nutrients. (2020) 12:805. 10.3390/nu12030805 32197411 PMC7146372

[28] American Diabetes Association. Diagnosis and classification of diabetes mellitus. Diabetes Care. (2014) 37(Suppl. 1):S81–90. 10.2337/dc14-S081 24357215

[29] Yuan X Liu T Wu L Zou ZY Li C. Validity of self-reported diabetes among middle-aged and older Chinese adults: the China Health and Retirement Longitudinal Study. BMJ Open. (2015) 5: e006633. 10.1136/bmjopen-2014-006633 25872937 PMC4401856

[30] Wu WT Li YJ Feng AZ Li L Huang T Xu AD Lyu J. Data mining in clinical big data: the frequently used databases, steps, and methodological models. Mil Med Res. (2021) 8:44. 10.1186/s40779-021-00338-z 34380547 PMC8356424

[31] Yuan YQ Li F Meng P You J Wu M Li SG. Gender difference on the association between dietary patterns and obesity in chinese middle-aged and elderly populations. Nutrients. (2016) 8:448. 10.3390/nu8080448 27455322 PMC4997363

[32] Song S Kim J Kim J. Gender differences in the association between dietary pattern and the incidence of hypertension in middle-aged and older adults. Nutrients. (2018) 10:252. 10.3390/nu10020252 29473892 PMC5852828

[33] Chen Z Zuurmond MG van der Schaft N Nano J Wijnhoven HAH Ikram MA. Plant versus animal based diets and insulin resistance, prediabetes and type 2 diabetes: the Rotterdam Study. Eur J Epidemiol. (2018) 33:883–93. 10.1007/s10654-018-0414-8 29948369 PMC6133017

[34] Yang X Li Y Wang C Mao Z Chen Y Ren P. Association of plant-based diet and type 2 diabetes mellitus in Chinese rural adults: the Henan Rural Cohort Study. J Diabetes Investig. (2021) 12:1569–76. 10.1111/jdi.13522 33559976 PMC8409831

[35] Qian F Liu G Hu FB Bhupathiraju SN Sun Q. Association between plant-based dietary patterns and risk of type 2 diabetes: a systematic review and meta-analysis. JAMA Intern Med. (2019) 179:1335–44. 10.1001/jamainternmed.2019.2195 31329220 PMC6646993

[36] Kim J Kim H Giovannucci EL. Quality of plant-based diets and risk of hypertension: a Korean genome and examination study. Eur J Nutr. (2021) 60:3841–51. 10.1007/s00394-021-02559-3 33864513

[37] Neuenschwander M Ballon A Weber KS Norat T Aune D Schwingshackl L. Role of diet in type 2 diabetes incidence: umbrella review of meta-analyses of prospective observational studies. BMJ. (2019) 366:l2368. 10.1136/bmj.l2368 31270064 PMC6607211

[38] van Baak MA. Nutrition as a link between obesity and cardiovascular disease: how can we stop the obesity epidemic? Thromb Haemost. (2013) 110:689–96. 10.1160/TH13-01-0045 23945609

[39] Im DS. FFA4 (GPR120) as a fatty acid sensor involved in appetite control, insulin sensitivity and inflammation regulation. Mol Aspects Med. (2018) 64:92–108. 10.1016/j.mam.2017.09.001 28887275

[40] Vučić Lovrenčić M Gerić M Košuta I Dragičević M Garaj-Vrhovac V Gajski G. Sex-specific effects of vegetarian diet on adiponectin levels and insulin sensitivity in healthy non-obese individuals. Nutrition. (2020) 79–80:110862. 10.1016/j.nut.2020.110862 32711387

[41] Robinson K Prins J Venkatesh B. Clinical review: adiponectin biology and its role in inflammation and critical illness. Crit Care. (2011) 15:221. 10.1186/cc10021 21586104 PMC3219307

[42] Schwingshackl L Schwedhelm C Hoffmann G Knüppel S Iqbal K Andriolo V. Food groups and risk of hypertension: a systematic review and dose-response meta-analysis of prospective studies. Adv Nutr. (2017) 8:793–803. 10.3945/an.117.017178 29141965 PMC5683007

[43] Yokoyama Y Nishimura K Barnard ND Takegami M Watanabe M Sekikawa A. Vegetarian diets and blood pressure: a meta-analysis. JAMA Intern Med. (2014) 174:577–87. 10.1001/jamainternmed.2013.14547 24566947

[44] McEvoy CT Temple N Woodside JV. Vegetarian diets, low-meat diets and health: a review. Public Health Nutr. (2012) 15:2287–94. 10.1017/S1368980012000936 22717188 PMC10271837

[45] Appleby PN Key TJ. The long-term health of vegetarians and vegans. Proc Nutr Soc. (2016) 75:287–93. 10.1017/S0029665115004334 26707634

[46] Lattimer JM Haub MD. Effects of dietary fiber and its components on metabolic health. Nutrients. (2010) 2:1266–89. 10.3390/nu2121266 22254008 PMC3257631

