# Supplementary material for: The Association Between Plant-Based Diet Indices and Obesity and Metabolic Diseases in Chinese Adults: Longitudinal Analyses From the China Health and Nutrition Survey
Source: Front Nutr. 2022 Jun 20;9:881901. doi: 10.3389/fnut.2022.881901 (PMC9251425; doi:10.3389/fnut.2022.881901)
Supplement: Supplementary file 1 [file Table_1.docx]

**S1 Table. Scoring system and classification of food items^a^.**

| Food groups | Items in the food frequency questionnaire | Food Codes (FD)^b^ | PDI | hPDI |
| --- | --- | --- | --- | --- |
| Healthy Plant Foods |  |  |  |  |
| Whole grains | Wheat, wheat germ flour, bran, black rice, brown rice, corn, barley, millet, yellow rice,cereal, oatmeal | 011101 =<FD=< 011208 or 012001=<FD=<012306 or 013101 =<FD=<013201 or 014101 =<FD=<019014 or FD=152102 or FD=152103 | Positive | Positive |
| Fruits | kernel fruits,berry fruit, citrus, tropical and subtropical fruits, melon, fruit juice, melons | 061101=<FD=<066206 or 162001 =<FD=<162028 | Positive | Positive |
| Vegetables | Root vegetables, fresh beans, eggplant, garlic and onion, young stems and leaves, aquatic vegetables, potatoes and taro, wild vegetables, mushrooms and algae | 041101=<FD=<048088 or 051001=<FD=<052011 or 021201=<FD=<021301 | Positive | Positive |
| Nuts | Peanuts/almonds/pine nuts | 071001=<FD=<072026 | Positive | Positive |
| Legumes | Non- fermented bean products : tofu, soy milk powder, mung bean, red bean, broad bean, other beans | 031101=<FD=<039902 | Positive | Positive |
| Tea and coffee | Coffee, tea | 166101=<FD=<166202 or 167016=<FD=<167018 | Positive | Positive |
| Less Healthy Plant Foods |  |  |  |  |
| Refined grains | Noodles,white rice, bread, biscuits, and other leisure foods, flour, instant noodles, starch,rice cake/rice cake soup, other rice cakes | 011301=<FD=<011503 or 012401=<FD=<012411 or FD=0132202 or FD=019201 or FD=152101 or 152104=<FD=<153002 or FD=153004 or 141001=<FD=<141037 or 022101=<FD=<022203 | Positive | Reverse |
| Potatoes | Potatoes, chips | 021101=<FD=<021108 or FD=153003 | Positive | Reverse |
| Sugar sweetened beverages | Carbonated beverages, solid beverages, milk beverages, plant protein beverages, other beverages. | 161001 =< FD=161008 or 163001=<FD=<165002 or 167001=<FD=<169013 | Positive | Reverse |
| Sweets and desserts | Cakes, desserts, mooncakes, sugars, preserves | 142101=<FD=<142333 or 181001=<FD=<184005 | Positive | Reverse |
| fermented food group | Bean sauce, peanut butter, salted vegetable, pickled vegetable (preserved in soy sauce or salt) | 203101=<FD=<205048 | Positive | Reverse |
| Animal Foods |  |  |  |  |
| Animal fat | Animal(pig,cattle and sheep) oils and fats | 191001=<FD=<191007 | Reverse | Reverse |
| Dairy | Milk, yogurt/yoplait, ice cream, cheese | 101101=<FD=<109009 | Reverse | Reverse |
| Eggs | Eggs/quail eggs | 111101=<FD=<114201 | Reverse | Reverse |
| Fish | Fish, shrimp, crab, shellfish and mollusc | 121101=<FD=<129302 | Reverse | Reverse |
| Meat | Subclasses of pig, sheep, donkey, horse, poultry, etc | 081101=<FD=<089006 or 091101=<FD=<099004 | Reverse | Reverse |

*^a^ The PDI and hPDI categorized food groups to “healthy plant foods,” “less healthy plant foods,” and “animal foods.” Positive indicates that higher intakes received higher scores. Reverse indicates that higher intakes received lower scores.*

*^B^ Food codes was derived from the Chinese food composition code list (2002/2004 edition).*

*PDI, overall plant-based diet index; hPDI, healthful plant-based diet index.*
