# Supplementary material for: The Association Between Plant-Based Diet Indices and Obesity and Metabolic Diseases in Chinese Adults: Longitudinal Analyses From the China Health and Nutrition Survey
Source: Front Nutr. 2022 Jun 20;9:881901. doi: 10.3389/fnut.2022.881901 (PMC9251425; doi:10.3389/fnut.2022.881901)
Supplement: Supplementary file 2 [file Table_2.docx]

**S2 Table. Hazard ratios (95% CI) for overweight/obesity, hypertension and type 2 diabetes according to quintile of the overall & healthful plant-based diet indices, stratified by sex**

|  | **Quintile 1** | **Quintile 2** | **Quintile 3** | **Quintile 4** | **Quintile 5** | **P-trend^a^** |
| --- | --- | --- | --- | --- | --- | --- |
| **OVERALL PLANT-BASED DIET INDEX** | | | | | | |
| **Overweight/Obesity** | | | | | | |
| Male | 1.00 | 0.79 (0.58, 1.08) | 0.82 (0.60, 1.12) | 0.73 (0.53, 1.01) | 0.76 (0.55, 1.05) | 0.08 |
| Female | 1.00 | 0.96 (0.69, 1.35) | 0.88 (0.63, 1.23) | 0.78 (0.55, 1.11) | 0.62 (0.42, 0.91) | <0.001 |
| **Hypertension** | | | | | | |
| Male | 1.00 | 0.92 (0.70, 1.20) | 0.79 (0.59, 1.04) | 0.99 (0.75, 1.30) | 0.65 (0.48, 0.88) | 0.01 |
| Female | 1.00 | 0.71 (0.53, 0.96) | 0.74 (0.56, 0.98) | 0.74 (0.55, 1.00) | 0.58 (0.41, 0.83) | <0.001 |
| **Type 2 diabetes mellitus** | | | | | | |
| Male | 1.00 | 0.91 (0.80, 1.04) | 0.90 (0.79, 1.02) | 0.89 (0.78, 1.01) | 0.82 (0.72, 0.94) | 0.005 |
| Female | 1.00 | 0.89 (0.77, 1.03) | 0.86 (0.75, 0.99) | 0.84 (0.73, 0.97) | 0.79 (0.68, 0.91) | 0.001 |
| **HEALTHFUL PLANT-BASED DIET INDEX** | | | | | | |
| **Overweight/Obesity** | | | | | | |
| Male | 1.00 | 0.84 (0.65, 1.14) | 0.76 (0.55, 1.05) | 0.91 (0.67, 1.25) | 0.83 (0.60, 1.14) | 0.39 |
| Female | 1.00 | 1.01 (0.74, 1.38) | 0.69 (0.47, 1.02) | 0.68 (0.46, 1.01) | 0.74 (0.53, 1.00) | 0.01 |
| **Hypertension** | | | | | | |
| Male | 1.00 | 1.11 (0.83, 1.48) | 1.15 (0.88, 1.51) | 1.02 (0.78, 1.36) | 0.87 (0.63, 1.20) | 0.39 |
| Female | 1.00 | 0.86 (0.64, 1.16) | 1.08 (0.78, 1.49) | 0.84 (0.60, 1.17) | 0.77 (0.57, 1.04) | 0.11 |
| **Type 2 diabetes mellitus** | | | | | | |
| Male | 1.00 | 0.92 (0.81, 1.05) | 0.89 (0.78, 1.01) | 0.89 (0.77, 1.01) | 0.84 (0.73, 0.98) | 0.02 |
| Female | 1.00 | 0.98 (0.85, 1.12) | 0.93 (0.80, 1.07) | 0.93 (0.79, 1.09) | 0.85 (0.73, 0.97) | 0.01 |
|  |  |  |  |  |  |  |

*Adjusted for age, urban and rural, total energy intake, education, physical activity, smoking status, alcohol drinking, baseline systolic and diastolic blood pressure, and BMI.*

*^a^ p-value when we assigned the median value to each quintile and entered this as a continuous variable in the model.*
