# Supplementary material for: The Association Between Plant-Based Diet Indices and Obesity and Metabolic Diseases in Chinese Adults: Longitudinal Analyses From the China Health and Nutrition Survey
Source: Front Nutr. 2022 Jun 20;9:881901. doi: 10.3389/fnut.2022.881901 (PMC9251425; doi:10.3389/fnut.2022.881901)
Supplement: Supplementary file 3 [file Table_3.docx]

**S3 Table. Characteristics of food type according to quintiles of plant-based diet indices^a^.**

| **Overweight/Obesity (N = 3795)** | | | | | |
| --- | --- | --- | --- | --- | --- |
|  | Quintile 1 | Quintile 2 | Quintile 3 | Quintile 4 | Quintile 5 |
| **Overall plant-based diet index** | | | | | |
| Total Energy Intake, kcal/d | 2204.4 (687.2) | 2240.8 (621.7) | 2272.1 (584.5) | 2265.4 (611.8) | 2237.5 (575.9) |
| Carbohydrate, kcal/d | 334.4 (118.5) | 339.5 (110.6) | 337.8 (102.2) | 332.3 (104.1) | 324.9 (107.8) |
| Protein, kcal/d | 66.2 (40.3) | 65.3 (35.2) | 66.2 (33.3) | 68.7 (36.8) | 70.4 (35.4) |
| Fat, kcal/d | 63.7 (27.3) | 66.7 (27.6) | 67.1 (22.4) | 68.1 (22.2) | 69.4 (23.2) |
| Healthful foods group |  |  |  |  |  |
| Whole grains, g/d | 99.1 (187.4) | 73.5 (159.3) | 66.1 (120.4) | 60.0 (114.5) | 58.0 (91.3) |
| Fruits, g/d | 10.0 (51.8) | 16.3 (74.4) | 26.3 (84.6) | 51.8 (123.1) | 118.2 (184.2) |
| Vegetables, g/d | 320.7 (170.6) | 367.4 (173.1) | 373.1 (180.0) | 369.3 (176.6) | 380.4 (166.7) |
| Nuts, g/d, g/d | 0.6 (4.9) | 1.6 (8.2) | 3.2 (13.8) | 5.0 (17.0) | 13.2 (31.2) |
| Legumes, g/d | 31.0 (57.7) | 45.3 (61.4) | 61.9 (67.8) | 70.8 (66.4) | 94.4 (79.0) |
| Tea and coffee, g/d | 0.0 (0.0) | 0.0 (0.0) | 0.4 (8.6) | 0.0 (0.0) | 1.6 (23.5) |
| Vegetable oil, g/d | 0.1 (1.3) | 0.1 (2.1) | 0.1 (0.8) | 0.2 (4.2) | 0.1 (2.5) |
| Unhealthful foods group |  |  |  |  |  |
| Refined grains, g/d | 331.6 (183.9) | 377.2 (167.4) | 388.6 (151.2) | 401.1 (167.0) | 409.3 (161.4) |
| Potatoes, g/d | 28.1 (76.0) | 34.3 (80.3) | 36.1 (61.0) | 43.9 (61.7) | 60.2 (72.6) |
| Sweetened beverages, g/d | 0.0 (0.0) | 0.2 (3.5) | 1.1 (12.6) | 0.7 (9.7) | 1.9 (14.4) |
| Sweets desserts, g/d | 0.2 (3.3) | 0.7 (6.3) | 1.1 (9.6) | 2.7 (13.1) | 6.9 (26.0) |
| Salty foods, g/d | 2.1 (11.2) | 7.1 (32.2) | 7.6 (17.6) | 10.5 (19.3) | 16.5 (27.1) |
| Animal foods group |  |  |  |  |  |
| Eggs, g/d | 19.2 (43.3) | 26.9 (41.1) | 31.3 (33.4) | 34.0 (38.0) | 36.2 (32.4) |
| Dairy, g/d | 3.07 (28.8) | 4.3 (34.2) | 10.1 (44.9) | 17.1 (56.9) | 29.4 (75.1) |
| Fish and seafood, g/d | 23.6 (57.5) | 33.1 (59.2) | 32.9 (51.2) | 45.2 (59.5) | 46.7 (52.8) |
| Meat, g/d | 92.2 (108.5) | 89.5 (80.8) | 86.0 (71.8) | 83.9 (66.6) | 79.3 (59.0) |
| Animal fat, g/d | 0.0 (0.0) | 0.0 (0.0) | 0.0 (0.0) | 0.0 (0.0) | 0.0 (0.0) |
| **Healthful plant-based diet index** | | | | | |
| Total Energy Intake, kcal/d | 2191.4 (625.1) | 2230.2 (628.7) | 2269.4 (625.1) | 2228.8 (607.4) | 2305.1 (591.9) |
| Carbohydrate, kcal/d | 348.6 (108.4) | 340.8 (111.9) | 331.4 (109.6) | 312.9 (99.1) | 337.1 (111.0) |
| Protein, kcal/d | 60.6 (34.3) | 63.9 (35.7) | 69.2 (34.9) | 73.6 (36.5) | 69.3 (38.0) |
| Fat, kcal/d | 62.6 (22.6) | 67.3 (29.8) | 67.3 (22.7) | 69.2 (23.7) | 68.5 (23.8) |
| Healthful foods group |  |  |  |  |  |
| Whole grains, g/d | 40.3 (116.1) | 66.0 (150.7) | 87.9 (172.7) | 91.3 (138.6) | 70.3 (106.8) |
| Fruits, g/d | 5.4 (23.9) | 14.5 (56.4) | 30.0 (89.2) | 53.1 (102.4) | 111.2 (201.2) |
| Vegetables, g/d | 353.4 (169.4) | 366.7 (182.0) | 369.8 (184.9) | 349.9 (157.8) | 375.7 (176.9) |
| Nuts, g/d, g/d | 0.5 (4.5) | 1.8 (8.9) | 3.0 (12.7) | 7.3 (20.5) | 10.2 (28.5) |
| Legumes, g/d | 27.8 (49.9) | 51.7 (64.3) | 58.4 (60.3) | 78.1 (72.8) | 84.3 (82.2) |
| Tea and coffee, g/d | 0.0 (0.0) | 0.2 (5.9) | 0.0 (0.0) | 0.3 (6.0) | 1.4 (22.4) |
| Vegetable oil, g/d | 0.0 (0.1) | 0.0 (1.6) | 0.0 (1.7) | 0.2 (3.0) | 0.2 (4.0) |
| Unhealthful foods group |  |  |  |  |  |
| Refined grains, g/d | 428.5 (175.9) | 383.6 (148.4) | 350.4 (141.3) | 319.1 (130.4) | 427.5 (205.6) |
| Potatoes, g/d | 14.7 (64.9) | 25.5 (64.9) | 33.8 (58.0) | 46.0 (69.8) | 81.1 (81.1) |
| Sweetened beverages, g/d | 0.0 (0.0) | 0.2 (4.3) | 1.7 (16.2) | 1.0 (11.1) | 0.7 (7.5) |
| Sweets desserts, g/d | 0.3 (4.9) | 0.6 (6.4) | 1.7 (12.9) | 3.8 (18.8) | 4.8 (19.1) |
| Salty foods, g/d | 8.0 (22.3) | 8.8 (35.0) | 8.5 (19.3) | 9.1 (17.9) | 9.1 (20.0) |
| Animal foods group |  |  |  |  |  |
| Eggs, g/d | 20.1 (49.1) | 25.7 (31.3) | 31.6 (30.0) | 34.7 (35.0) | 35.5 (41.4) |
| Dairy, g/d | 0.8 (13.0) | 3.9 (33.5) | 9.3 (44.0) | 22.3 (73.0) | 25.5 (62.7) |
| Fish and seafood, g/d | 26.3 (56.7) | 34.4 (58.9) | 41.3 (55.9) | 44.5 (58.7) | 35.0 (52.4) |
| Meat, g/d | 87.5 (97.2) | 87.8 (76.9) | 90.6 (74.2) | 88.0 (72.6) | 77.8 (69.9) |
| Animal fat, g/d | 0.0 (0.0) | 0.0 (0.0) | 0.0 (0.0) | 0.0 (0.0) | 0.0 (0.0) |
|  |  |  |  |  |  |
| **Incident Hypertension (N = 4775)** | | | | | |
|  | Quintile 1 | Quintile 2 | Quintile 3 | Quintile 4 | Quintile 5 |
| **Overall plant-based diet index** | | | | | |
| Total Energy Intake, kcal/d | 2202.2 (663.1) | 2205.7 (604.5) | 2282.9 (617.7) | 2293.0 (621.8) | 2266.2 (559.9) |
| Carbohydrate, kcal/d | 337.7 (118.1) | 330.3 (110.1) | 336.9 (109.2) | 332.8 (107.2) | 329.9 (103.5) |
| Protein, kcal/d | 65.8 (39.3) | 65.9 (34.6) | 67.6 (34.6) | 70.3 (37.4) | 71.8 (35.2) |
| Fat, kcal/d | 64.9 (26.8) | 65.0 (22.6) | 67.7 (23.6) | 69.2 (23.5) | 70.6 (22.7) |
| Healthful foods group |  |  |  |  |  |
| Whole grains, g/d | 104.1 (199.7) | 71.4 (147.5) | 70.8 (140.8) | 66.9 (127.2) | 67.5 (102.5) |
| Fruits, g/d | 10.7 (68.3) | 16.0 (54.2) | 23.4 (65.7) | 53.4 (123.1) | 129.0 (204.7) |
| Vegetables, g/d | 362.0 (163.9) | 351.7 (175.4) | 380.1 (190.3) | 364.1 (170.1) | 380.7 (174.3) |
| Nuts, g/d, g/d | 0.8 (7.2) | 1.8 (9.1) | 3.6 (14.1) | 4.9 (17.4) | 12.7 (29.3) |
| Legumes, g/d | 32.4 (58.1) | 47.4 (56.8) | 63.5 (70.0) | 69.6 (61.9) | 93.6 (79.6) |
| Tea and coffee, g/d | 0.0 (0.0) | 0.0 (0.0) | 0.3 (6.7) | 0.4 (8.7) | 2.2 (34.4) |
| Vegetable oil, g/d | 0.1 (2.5) | 0.1 (2.4) | 0.2 (3.3) | 0.3 (5.2) | 0.2 (3.3) |
| Unhealthful foods group |  |  |  |  |  |
| Refined grains, g/d | 330.2 (181.7) | 368.6 (174.7) | 387.0 (162.1) | 397.3 (169.5) | 411.5 (169.1) |
| Potatoes, g/d | 31.1 (76.6) | 38.3 (77.5) | 36.0 (65.8) | 49.7 (65.8) | 61.9 (70.3) |
| Sweetened beverages, g/d | 0.0 (2.5) | 0.5 (8.8) | 0.2 (5.6) | 0.9 (13.1) | 1.7 (13.6) |
| Sweets desserts, g/d | 0.2 (3.0) | 0.8 (7.2) | 1.1 (9.9) | 3.1 (13.6) | 7.1 (25.3) |
| Salty foods, g/d | 2.3 (10.5) | 5.9 (20.1) | 7.4 (19.3) | 9.8 (20.0) | 15.4 (25.3) |
| Animal foods group |  |  |  |  |  |
| Eggs, g/d | 20.1 (42.6) | 27.2 (34.7) | 31.9 (36.2) | 35.1 (34.3) | 36.5 (32.9) |
| Dairy, g/d | 1.5 (21.0) | 6.9 (38.1) | 9.6 (44.2) | 15.4 (55.3) | 28.0 (72.5) |
| Fish and seafood, g/d | 22.5 (53.6) | 33.5 (59.5) | 33.7 (54.7) | 45.2 (62.0) | 43.7 (50.6) |
| Meat, g/d | 89.2 (102.1) | 90.9 (83.8) | 86.5 (76.3) | 87.3 (74.0) | 79.6 (59.5) |
| Animal fat, g/d | 0.0 (0.0) | 0.0 (0.0) | 0.0 (0.2) | 0.0 (0.0) | 0.0 (0.0) |
| **Healthful plant-based diet index** | | | | | |
| Total Energy Intake, kcal/d | 2223.6 (620.1) | 2217.9 (645.6) | 2288.0 (649.4) | 2241.2 (601.8) | 2294.3 (553.0) |
| Carbohydrate, kcal/d | 358.2 (111.6) | 342.1 (114.8) | 332.8 (116.5) | 317.4 (103.6) | 311.1 (93.4) |
| Protein, kcal/d | 61.0 (34.7) | 62.5 (35.7) | 69.0 (34.0) | 71.7 (36.5) | 80.2 (38.2) |
| Fat, kcal/d | 64.3 (25.7) | 65.6 (22.4) | 67.3 (22.6) | 68.5 (23.6) | 72.7 (24.9) |
| Healthful foods group |  |  |  |  |  |
| Whole grains, g/d | 38.1 (120.6) | 61.7 (138.1) | 79.5 (162.3) | 109.1 (182.9) | 107.4 (122.3) |
| Fruits, g/d | 5.2 (28.7) | 20.0 (77.2) | 36.9 (104.8) | 59.4 (151.4) | 123.6 (181.3) |
| Vegetables, g/d | 358.7 (177.9) | 363.3 (179.1) | 373.8 (187.8) | 349.5 (177.3) | 352.0 (151.7) |
| Nuts, g/d, g/d | 0.6 (5.5) | 1.8 (10.6) | 2.9 (11.0) | 5.5 (17.5) | 14.3 (31.5) |
| Legumes, g/d | 29.0 (49.3) | 52.4 (58.4) | 62.8 (71.0) | 73.1 (71.2) | 94.8 (77.6) |
| Tea and coffee, g/d | 0.0 (0.0) | 0.1 (5.0) | 0.4 (4.1) | 0.1 (2.7) | 2.9 (37.5) |
| Vegetable oil, g/d | 0.0 (0.9) | 0.1 (1.9) | 0.1 (2.1) | 0.6 (6.1) | 0.3 (3.9) |
| Unhealthful foods group |  |  |  |  |  |
| Refined grains, g/d | 451.6 (192.1) | 407.7 (178.8) | 374.8 (160.9) | 328.5 (147.5) | 303.0 (133.0) |
| Potatoes, g/d | 32.4 (81.9) | 37.0 (72.8) | 42.0 (71.5) | 50.7 (66.2) | 57.0 (62.6) |
| Sweetened beverages, g/d | 0.0 (0.0) | 0.2 (5.3) | 1.5 (15.5) | 0.6 (8.7) | 1.4 (13.3) |
| Sweets desserts, g/d | 0.2 (4.1) | 0.7 (10.6) | 1.6 (10.1) | 3.4 (19.4) | 7.1 (19.8) |
| Salty foods, g/d | 7.0 (23.7) | 6.9 (20.5) | 8.4 (18.9) | 8.9 (19.5) | 9.0 (14.3) |
| Animal foods group |  |  |  |  |  |
| Eggs, g/d | 19.6 (46.4) | 27.0 (32.2) | 34.5 (31.4) | 35.5 (37.2) | 39.0 (29.7) |
| Dairy, g/d | 0.6 (11.9) | 4.2 (31.4) | 9.8 (44.5) | 17.4 (65.5) | 32.7 (70.5) |
| Fish and seafood, g/d | 24.5 (55.3) | 31.1 (57.1) | 39.1 (56.7) | 39.3 (57.1) | 47.0 (55.5) |
| Meat, g/d | 83.9 (92.6) | 83.4 (83.7) | 91.8 (80.3) | 83.0 (74.0) | 94.7 (69.0) |
| Animal fat, g/d | 0.0 (0.0) | 0.0 (0.0) | 0.0 (0.0) | 0.0 (0.0) | 0.0 (0.2) |
|  |  |  |  |  |  |
| **Incident Type 2 diabetes mellitus (N = 8211)** | | | | | |
|  | Quintile 1 | Quintile 2 | Quintile 3 | Quintile 4 | Quintile 5 |
| **Overall plant-based diet index** | | | | | |
| Total Energy Intake, kcal/d | 2168.0 (629.6) | 2168.7 (581.6) | 2229.4 (568.5) | 2203.4 (543.1) | 2246.7 (542.7) |
| Carbohydrate, kcal/d | 328.4 (119.6) | 323.1 (107.4) | 327.9 (105.1) | 320.0 (101.2) | 320.9 (106.0) |
| Protein, kcal/d | 66.5 (40.7) | 66.3 (36.4) | 68.5 (36.6) | 70.3 (35.7) | 72.4 (35.9) |
| Fat, kcal/d | 63.8 (26.4) | 64.8 (23.7) | 67.2 (23.5) | 67.8 (22.0) | 69.6 (23.0) |
| Healthful foods group |  |  |  |  |  |
| Whole grains, g/d | 102.0 (194.1) | 72.4 (141.0) | 69.8 (119.4) | 62.2 (110.4) | 68.7 (102.6) |
| Fruits, g/d | 9.9 (64.1) | 17.9 (58.1) | 35.5 (91.6) | 67.4 (143.2) | 130.9 (179.2) |
| Vegetables, g/d | 324.3 (166.0) | 349.9 (173.3) | 365.8 (168.2) | 356.8 (155.8) | 377.7 (165.1) |
| Nuts, g/d, g/d | 0.6 (5.3) | 2.2 (12.7) | 3.0 (11.9) | 5.1 (17.5) | 14.1 (33.7) |
| Legumes, g/d | 32.8 (56.9) | 49.5 (61.8) | 64.5 (64.3) | 74.3 (65.8) | 93.0 (73.5) |
| Tea and coffee, g/d | 0.0 (0.0) | 0.0 (0.0) | 0.1 (4.1) | 0.2 (6.3) | 2.2 (32.3) |
| Vegetable oil, g/d | 0.1 (2.0) | 0.2 (2.8) | 0.2 (2.7) | 0.2 (3.6) | 0.2 (3.2) |
| Unhealthful foods group |  |  |  |  |  |
| Refined grains, g/d | 324.5 (172.5) | 360.9 (160.9) | 381.0 (164.1) | 384.2 (153.5) | 404.5 (160.8) |
| Potatoes, g/d | 29.3 (71.9) | 38.2 (71.9) | 40.8 (63.4) | 48.9 (68.6) | 59.7 (67.2) |
| Sweetened beverages, g/d | 0.0 (1.6) | 0.5 (8.1) | 0.8 (12.0) | 0.5 (8.7) | 1.7 (13.4) |
| Sweets desserts, g/d | 0.3 (5.5) | 1.0 (7.9) | 1.5 (10.5) | 3.5 (14.6) | 7.5 (23.6) |
| Salty foods, g/d | 2.4 (12.0) | 5.8 (17.1) | 7.8 (27.8) | 10.3 (18.9) | 16.6 (29.0) |
| Animal foods group |  |  |  |  |  |
| Eggs, g/d | 22.7 (43.9) | 29.6 (41.2) | 33.8 (37.1) | 36.1 (30.0) | 38.3 (31.6) |
| Dairy, g/d | 3.7 (30.9) | 6.5 (35.4) | 15.6 (59.6) | 22.3 (62.6) | 35.4 (74.7) |
| Fish and seafood, g/d | 25.7 (56.9) | 35.5 (83.1) | 37.3 (56.2) | 46.2 (56.5) | 46.7 (49.9) |
| Meat, g/d | 92.7 (101.2) | 95.2 (82.6) | 88.4 (70.4) | 88.0 (67.1) | 80.7 (59.1) |
| Animal fat, g/d | 0.0 (0.0) | 0.0 (0.0) | 0.0 (3.1) | 0.0 (3.3) | 0.0 (0.0) |
| **Healthful plant-based diet index** | | | | | |
| Total Energy Intake, kcal/d | 2187.3 (602.0) | 2158.2 (597.5) | 2209.8 (575.0) | 2217.3 (566.3) | 2241.3 (522.7) |
| Carbohydrate, kcal/d | 353.8 (114.5) | 332.2 (113.5) | 323.5 (109.0) | 311.0 (99.0) | 298.3 (93.2) |
| Protein, kcal/d | 59.4 (36.6) | 62.7 (35.1) | 67.9 (34.5) | 73.9 (38.9) | 80.6 (37.1) |
| Fat, kcal/d | 62.8 (23.5) | 64.7 (25.1) | 66.3 (22.3) | 68.5 (23.3) | 70.9 (24.2) |
| Healthful foods group |  |  |  |  |  |
| Whole grains, g/d | 38.4 (115.4) | 57.1 (132.0) | 77.8 (150.3) | 99.4 (156.0) | 103.0 (116.8) |
| Fruits, g/d | 7.5 (45.0) | 18.1 (70.2) | 35.7 (95.2) | 65.3 (134.7) | 137.4 (184.0) |
| Vegetables, g/d | 354.7 (183.2) | 354.9 (165.6) | 363.4 (178.2) | 350.0 (159.3) | 348.9 (142.5) |
| Nuts, g/d, g/d | 0.3 (4.2) | 1.7 (8.7) | 3.0 (13.5) | 5.8 (18.6) | 14.7 (34.6) |
| Legumes, g/d | 31.5 (53.0) | 51.6 (60.8) | 62.5 (64.6) | 77.1 (70.2) | 90.5 (73.4) |
| Tea and coffee, g/d | 0.0 (0.0) | 0.0 (4.0) | 0.0 (0.1) | 0.0 (2.9) | 2.6 (33.9) |
| Vegetable oil, g/d | 0.0 (0.9) | 0.1 (2.0) | 0.1 (2.1) | 0.3 (3.8) | 0.3 (4.5) |
| Unhealthful foods group |  |  |  |  |  |
| Refined grains, g/d | 451.4 (184.3) | 398.7 (162.3) | 368.5 (155.7) | 332.4 (148.5) | 297.0 (121.5) |
| Potatoes, g/d | 32.2 (75.7) | 36.1 (72.6) | 43.6 (68.1) | 49.8 (67.9) | 54.8 (58.9) |
| Sweetened beverages, g/d | 0.1 (5.0) | 0.2 (4.9) | 1.0 (12.4) | 0.4 (7.1) | 1.7 (14.8) |
| Sweets desserts, g/d | 0.2 (4.1) | 0.8 (10.4) | 2.0 (12.8) | 3.2 (15.8) | 7.7 (20.7) |
| Salty foods, g/d | 7.5 (31.1) | 7.2 (19.2) | 8.9 (23.2) | 8.9 (18.3) | 9.8 (16.3) |
| Animal foods group |  |  |  |  |  |
| Eggs, g/d | 21.0 (47.6) | 28.2 (34.0) | 34.3 (38.1) | 37.5 (35.2) | 39.1 (27.8) |
| Dairy, g/d | 1.4 (20.3) | 5.0 (32.4) | 11.5 (51.0) | 20.6 (63.6) | 46.8 (81.4) |
| Fish and seafood, g/d | 24.9 (55.4) | 33.2 (82.3) | 40.1 (58.8) | 43.7 (57.4) | 49.6 (52.2) |
| Meat, g/d | 86.1 (93.3) | 87.9 (79.9) | 88.3 (73.1) | 88.5 (72.2) | 96.3 (68.2) |
| Animal fat, g/d | 0.0 (0.0) | 0.0 (3.2) | 0.0 (2.9) | 0.0 (0.0) | 0.0 (0.1) |

*a: Values are expressed as mean ± SD.*
