# Supplementary material for: The Association Between Plant-Based Diet Indices and Obesity and Metabolic Diseases in Chinese Adults: Longitudinal Analyses From the China Health and Nutrition Survey
Source: Front Nutr. 2022 Jun 20;9:881901. doi: 10.3389/fnut.2022.881901 (PMC9251425; doi:10.3389/fnut.2022.881901)
Supplement: Supplementary file 4 [file Presentation_1.PDF]

CHINA ECONOMIC, POPULATION, NUTRITION, AND HEALTH SURVEY

2006 DIETARY QUESTIONNAIRE

Province: 21 Liaoning 23 Heilongjiang 32 Jiangsu 37 Shandong 41 Henan ☐☐T1
42 Hubei 43 Hunan 45 Guangxi 52 Guizhou

Urban Site: 1 Rural Site: 2 ☐T2

Urban	Rural	
City: __________	County: ___________	☐T3
1 First city	1 First county	
2 Second city	2 Second county	
	3 Third county	
	4 Fourth county	

Urban	Rural	
Neighborhood: _____________	Village (Town): ___________	☐☐T4
01 First [urban] neighborhood	01 County town neighborhood	
02 Second [urban] neighborhood	02 First village	
03 Third suburban village (neighborhood)	03 Second village	
04 Fourth suburban village (neighborhood)	04 Third village	
05 Fifth [urban] neighborhood	05 County town neighborhood	
06 Sixth [urban] neighborhood	06 Fourth village	
07 Seventh suburban village (neighborhood)	07 Fifth village	
08 Eighth suburban village (neighborhood)	08 Sixth village	
09 Ninth [urban] neighborhood	09 County town neighborhood	
10 Tenth [urban] neighborhood	10 Seventh village	
11 Eleventh suburban village (neighborhood)	11 Eighth village	
12 Twelfth suburban village (neighborhood)	12 Ninth village	

Household Number: ___________ ☐☐☐T5

Interview Date: ____Year _____Month ____Day ☐☐☐☐☐☐☐☐T7

Completion Evaluation: 1 Good 2 OK 3 Poor ☐CO

Interviewer Name: ________ Number: __________ ☐☐T6c

Supervisor Name: _________ Number: __________ ☐☐T6d

Table 1. 3-Day Record of Household Food Consumption (gm)

Household ID: ☐☐ Province (T1) ☐ Site (T2) ☐ City/County (T3) ☐☐ Neighborhood/Village (T4) ☐☐☐ Household (T5)

			Day 1		Day 2		Day 3		Total				
1<br>Food Code	2<br>Food Name	3<br>Initial Amount on Hand	4<br>Amount Purchased or Grown	5<br>Amount Discarded	6<br>Amount Purchased or Grown	7<br>Amount Discarded	8<br>Amount Purchased or Grown	9<br>Amount Discarded	10<br>Amount Purchased or Grown	11<br>Amount Discarded	12<br>Total Remaining	13<br>Actual Consumption	14<br>Consumption per Person per Day
**V14**<br>_ _ _ _ _ _		**V15a**<br>_ _ _ _ _	**V24_16a**<br>_ _ _ _ _ _	**V25_16a**<br>_ _ _ _	**V24_17a**<br>_ _ _ _ _ _	**V25_17a**<br>_ _ _ _	**V24_18a**<br>_ _ _ _ _ _	**V25_18a**<br>_ _ _ _	**V24_20a**<br>_ _ _ _ _ _	**V25_20a**<br>_ _ _ _	**V21a**<br>_ _ _ _ _ _	**V22a**<br>_ _ _ _ _ _	**V23a**<br>_ _ _ _ _
_ _ _ _ _ _		_ _ _ _ _	_ _ _ _ _ _	_ _ _ _	_ _ _ _ _ _	_ _ _ _	_ _ _ _ _ _	_ _ _ _	_ _ _ _ _ _	_ _ _ _	_ _ _ _ _ _	_ _ _ _ _ _	_ _ _ _ _
_ _ _ _ _ _		_ _ _ _ _	_ _ _ _ _ _	_ _ _ _	_ _ _ _ _ _	_ _ _ _	_ _ _ _ _ _	_ _ _ _	_ _ _ _ _ _	_ _ _ _	_ _ _ _ _ _	_ _ _ _ _ _	_ _ _ _ _
_ _ _ _ _ _		_ _ _ _ _	_ _ _ _ _ _	_ _ _ _	_ _ _ _ _ _	_ _ _ _	_ _ _ _ _ _	_ _ _ _	_ _ _ _ _ _	_ _ _ _	_ _ _ _ _ _	_ _ _ _ _ _	_ _ _ _ _
_ _ _ _ _ _		_ _ _ _ _	_ _ _ _ _ _	_ _ _ _	_ _ _ _ _ _	_ _ _ _	_ _ _ _ _ _	_ _ _ _	_ _ _ _ _ _	_ _ _ _	_ _ _ _ _ _	_ _ _ _ _ _	_ _ _ _ _
_ _ _ _ _ _		_ _ _ _ _	_ _ _ _ _ _	_ _ _ _	_ _ _ _ _ _	_ _ _ _	_ _ _ _ _ _	_ _ _ _	_ _ _ _ _ _	_ _ _ _	_ _ _ _ _ _	_ _ _ _ _ _	_ _ _ _ _
_ _ _ _ _ _		_ _ _ _ _	_ _ _ _ _ _	_ _ _ _	_ _ _ _ _ _	_ _ _ _	_ _ _ _ _ _	_ _ _ _	_ _ _ _ _ _	_ _ _ _	_ _ _ _ _ _	_ _ _ _ _ _	_ _ _ _ _
_ _ _ _ _ _		_ _ _ _ _	_ _ _ _ _ _	_ _ _ _	_ _ _ _ _ _	_ _ _ _	_ _ _ _ _ _	_ _ _ _	_ _ _ _ _ _	_ _ _ _	_ _ _ _ _ _	_ _ _ _ _ _	_ _ _ _ _
_ _ _ _ _ _		_ _ _ _ _	_ _ _ _ _ _	_ _ _ _	_ _ _ _ _ _	_ _ _ _	_ _ _ _ _ _	_ _ _ _	_ _ _ _ _ _	_ _ _ _	_ _ _ _ _ _	_ _ _ _ _ _	_ _ _ _ _
_ _ _ _ _ _		_ _ _ _ _	_ _ _ _ _ _	_ _ _ _	_ _ _ _ _ _	_ _ _ _	_ _ _ _ _ _	_ _ _ _	_ _ _ _ _ _	_ _ _ _	_ _ _ _ _ _	_ _ _ _ _ _	_ _ _ _ _
_ _ _ _ _ _		_ _ _ _ _	_ _ _ _ _ _	_ _ _ _	_ _ _ _ _ _	_ _ _ _	_ _ _ _ _ _	_ _ _ _	_ _ _ _ _ _	_ _ _ _	_ _ _ _ _ _	_ _ _ _ _ _	_ _ _ _ _
_ _ _ _ _ _		_ _ _ _ _	_ _ _ _ _ _	_ _ _ _	_ _ _ _ _ _	_ _ _ _	_ _ _ _ _ _	_ _ _ _	_ _ _ _ _ _	_ _ _ _	_ _ _ _ _ _	_ _ _ _ _ _	_ _ _ _ _
_ _ _ _ _ _		_ _ _ _ _	_ _ _ _ _ _	_ _ _ _	_ _ _ _ _ _	_ _ _ _	_ _ _ _ _ _	_ _ _ _	_ _ _ _ _ _	_ _ _ _	_ _ _ _ _ _	_ _ _ _ _ _	_ _ _ _ _

[2.] Remember to include oil and condiments (salt, sugar, sauces, etc.) in the table. Please record oil and condiments first.

Formulas:

10. Total Amount Purchased or Grown: V24_20a = V24_16a + V24_17a + V24_18a
11. Total Amount Discarded: V25_20a = V25_16a + V25_17a + V25_18a
13. Actual Consumption = Initial Amount on Hand + Total Amount Purchased or Grown - Total Amount Discarded - Total Remaining

    V22a = V15a + V24_20a - V25_20a - V21a
14. Consumption per Person per Day = Actual Consumption / Total Household Person-Days

    V23a = V22a / item 26 from Table 2

Table 2. 3-Day Record of Household Meals Per Person Per Day

Household ID: ☐☐ Province (T1) ☐ Site (T2) ☐ City/County (T3) ☐☐ Neighborhood/Village (T4) ☐☐☐ Household (T5)

					Ate Meal At Home?																Typical Meal Proportions			
					Day 1				Day 2				Day 3				Total							
1 Line Number	2 Name	3 Age (years)	4 Sex	5 Activity Level	6 B	7 L	8 D	9 S	10 B	11 L	12 D	13 S	14 B	15 L	16 D	17 S	18 B	19 L	20 D	21 S	22 B	23 L	24 D	25 Number of Person-Days
**A1**		**V26**	**V27**	**V29**	**V3036**	**V3037**	**V3038**	**V3039**	**V3136**	**V3137**	**V3138**	**V3139**	**V3236**	**V3237**	**V3238**	**V3239**	**V3436**	**V3437**	**V3438**	**V3439**	**V44**	**V45**	**V46**	**V35**
_ _ _		_ _ _																			_ . _ _	_ . _ _	_ . _ _	_ . _ _
_ _ _		_ _ _																			_ . _ _	_ . _ _	_ . _ _	_ . _ _
_ _ _		_ _ _																			_ . _ _	_ . _ _	_ . _ _	_ . _ _
_ _ _		_ _ _																			_ . _ _	_ . _ _	_ . _ _	_ . _ _
_ _ _		_ _ _																			_ . _ _	_ . _ _	_ . _ _	_ . _ _
_ _ _		_ _ _																			_ . _ _	_ . _ _	_ . _ _	_ . _ _
_ _ _		_ _ _																			_ . _ _	_ . _ _	_ . _ _	_ . _ _
_ _ _		_ _ _																			_ . _ _	_ . _ _	_ . _ _	_ . _ _
_ _ _		_ _ _																			_ . _ _	_ . _ _	_ . _ _	_ . _ _
_ _ _		_ _ _																			_ . _ _	_ . _ _	_ . _ _	_ . _ _

Codes:
1. Use -91 through -99 as line numbers for guests
4. Sex:
   - 1 male
   - 2 female
5. Activity level:
   - 1 very light physical activity (working in a sitting position, e.g., office worker, watch repairer, etc.)
   - 2 light physical activity (working in standing position, e.g., salesperson, laboratory technician, teacher, etc.)
   - 3 moderate physical activity (student, driver, electrician, metal worker, etc.)
   - 4 heavy physical activity (farmer, dancer, steel worker, athlete, etc.)
   - 5 very heavy physical activity (loader, logger, miner, stonecutter, etc.)
   - 6 no working ability (under age 7)

6-17. Ate meal at home?
- 0 ate meal away from home
- 1 ate meal at home
- \- meal not eaten at all

- B breakfast
- L lunch
- D dinner
- S snack

26 Total Household Person-Days

_ _ . _ _

Table 3. Consumption of Snacks and Beverages

Household ID: ☐☐ Province (T1) ☐ Site (T2) ☐ City/County (T3) ☐☐ Neighborhood/Village (T4) ☐☐☐ HH (T5)

Name: ______________ Line Number: _________ ☐☐☐A1

Day	1<br>Item<br>Number	2<br>Meal	3<br>Ingredient<br>Name	4<br>Ingredient<br>Code	5<br>Amount<br>(gm)	6<br>Meal<br>Location	7<br>Preparation<br>Method
		**V40**		**V14b**	**V39a**	**V41**	**V42**
1	1	_		_ _ _ _ _ _	_ _ _ _	_	_
1	**2**	_		_ _ _ _ _ _	_ _ _ _	_	_
1	**3**	_		_ _ _ _ _ _	_ _ _ _	_	_
1	**4**	_		_ _ _ _ _ _	_ _ _ _	_	_
1	**5**	_		_ _ _ _ _ _	_ _ _ _	_	_
1	**6**	_		_ _ _ _ _ _	_ _ _ _	_	_
1	**7**	_		_ _ _ _ _ _	_ _ _ _	_	_
1	8	_		_ _ _ _ _ _	_ _ _ _	_	_
2	9	_		_ _ _ _ _ _	_ _ _ _	_	_
2	10	_		_ _ _ _ _ _	_ _ _ _	_	_
2	11	_		_ _ _ _ _ _	_ _ _ _	_	_
2	12	_		_ _ _ _ _ _	_ _ _ _	_	_
2	13	_		_ _ _ _ _ _	_ _ _ _	_	_
2	14	_		_ _ _ _ _ _	_ _ _ _	_	_
2	15	_		_ _ _ _ _ _	_ _ _ _	_	_
2	16	_		_ _ _ _ _ _	_ _ _ _	_	_
3	17	_		_ _ _ _ _ _	_ _ _ _	_	_
3	18	_		_ _ _ _ _ _	_ _ _ _	_	_
3	19	_		_ _ _ _ _ _	_ _ _ _	_	_
3	20	_		_ _ _ _ _ _	_ _ _ _	_	_
3	21	_		_ _ _ _ _ _	_ _ _ _	_	_
3	22	_		_ _ _ _ _ _	_ _ _ _	_	_
3	23	_		_ _ _ _ _ _	_ _ _ _	_	_
3	24	_		_ _ _ _ _ _	_ _ _ _	_	_

Codes:
2. 2 morning snack; 4 afternoon snack; 6 evening snack
6. 1 at home; 2 at school or work unit; 3 restaurant or food stand; 4 relative's or friend's house; 5 nursery school; 6 festival/celebration; 7 other
7. 1 boiled; 2 stir-fried; 3 deep-fried; 4 steamed; 5 griddle; 6 cooked food; 7 baked; 8 eaten raw; 9 other

Table 4. Individual Record of Daily Food

Household ID: ☐☐ Province (T1) ☐ Site (T2) ☐ City/County (T3) ☐☐ Neighborhood/Village (T4) ☐☐☐

Name:__________ Line Number:___________ ☐☐☐A1

Interview Day: 1. Day 1 2. Day 2 3. Day 3 ☐VD

Person-Day ☐.☐V35a

Interview Date: _____Year ______Month _______Day ☐☐☐☐☐☐☐☐ T7

1<br>Item<br>Number	2<br>Meal	3<br>Ingredient<br>Name	4<br>Ingredient Code	5<br>Amount<br>(gm)	6<br>Meal<br>Location	7<br>Preparation<br>Method	8<br>Preparation<br>Location
1	**V40**<br>_		**V14b**<br>_ _ _ _ _ _	**V39a**<br>_ _ _ _	**V41**<br>_	**V42**<br>_	**V43**<br>_
2	_		_ _ _ _ _ _	_ _ _ _	_	_	_
3	_		_ _ _ _ _ _	_ _ _ _	_	_	_
4	_		_ _ _ _ _ _	_ _ _ _	_	_	_
5	_		_ _ _ _ _ _	_ _ _ _	_	_	_
6	_		_ _ _ _ _ _	_ _ _ _	_	_	_
7	_		_ _ _ _ _ _	_ _ _ _	_	_	_
8	_		_ _ _ _ _ _	_ _ _ _	_	_	_
9	_		_ _ _ _ _ _	_ _ _ _	_	_	_
10	_		_ _ _ _ _ _	_ _ _ _	_	_	_
11	_		_ _ _ _ _ _	_ _ _ _	_	_	_
12	_		_ _ _ _ _ _	_ _ _ _	_	_	_
13	_		_ _ _ _ _ _	_ _ _ _	_	_	_
14	_		_ _ _ _ _ _	_ _ _ _	_	_	_
15	_		_ _ _ _ _ _	_ _ _ _	_	_	_
16	_		_ _ _ _ _ _	_ _ _ _	_	_	_
17	_		_ _ _ _ _ _	_ _ _ _	_	_	_
18	_		_ _ _ _ _ _	_ _ _ _	_	_	_
19	_		_ _ _ _ _ _	_ _ _ _	_	_	_
20	_		_ _ _ _ _ _	_ _ _ _	_	_	_

Codes:
2. 1 breakfast 2 morning snack 3 lunch 4 afternoon snack 5 dinner 6 evening snack
6. 1 at home 2 at school or work unit 3 restaurant or food stand 4 relative's or friend's house 5 nursery school 6 festival/celebration 7 other
7. 1 boiled 2 stir-fried 3 deep-fried 4 steamed 5 griddle 6 cooked food 7 baked 8 eaten raw 9 other
8. 1 at home 2 at school or work unit 3 restaurant or food stand 4 relative's or friend's house 5 nursery school 6 festival/celebration 7 other
